# Age-driven shifts in T and NK cell responses amplify inflammation and coagulopathy during viral infection in mice and humans

**DOI:** 10.3389/fimmu.2026.1712726

**Published:** 2026-02-09

**Authors:** Craig P. Collins, Cordelia Dunai, Logan V. Vick, Lam T. Khuat, Alexander Merleev, Eunju (April) Choi, Jonathan Lam, Emanual Maverakis, Arta M. Monjazeb, Dan L. Longo, Nicole Baumgarth, Robert J. Canter, William J. Murphy

**Affiliations:** 1 University of California, Davis, School of Medicine, Sacramento, CA, United States; 2 Department of Pathology, Microbiology and Immunology, School of Veterinary Medicine, Davis, CA, United States; 3 Ragon Institute of Mass General and Massachusetts Institute of Technology (MIT), Cambridge, MA, United States; 4 Department of Radiation Oncology, University of California, Davis Comprehensive Cancer Center, School of Medicine, Sacramento, CA, United States; 5 Department of Medicine, Harvard Medical School, Boston, MA, United States; 6 Johns Hopkins Bloomberg School of Public Health, Department of Molecular Microbiology and Immunology, Lyme and Tickborne Diseases Research and Education Institute, Baltimore, MD, United States; 7 Division of Surgical Oncology, Department of Surgery, University of California, Davis Comprehensive Cancer Center, School of Medicine, Sacramento, CA, United States; 8 Department of Internal Medicine, Division of Hematology and Oncology, University of California, Davis, School of Medicine, Sacramento, CA, United States

**Keywords:** ageing, aging, coagulopathy, NK cells, T cells, viral immune response

## Abstract

**Introduction:**

Advanced age is associated with increased morbidity and mortality following acute viral infections, including SARS-CoV-2. Despite this, most preclinical models rely on young animals and fail to account for age-related immune remodeling. How advanced aging alters antiviral immune responses and contributes to immune-mediated pathology remains incompletely understood.

**Methods:**

Young (2–6 months), aged (15–18 months), and advanced aged (20–29 months) mice were infected with murine cytomegalovirus (MCMV) or influenza virus. Survival, viral burden, cytokine production, immune cell phenotypes, and tissue pathology were assessed using flow cytometry, histology, serum cytokine analysis, and RNA sequencing. Mouse findings were compared with publicly available transcriptomic datasets from SARS-CoV-2–infected human cohorts across age groups.

**Results:**

Advanced aged mice exhibited markedly increased mortality and organ pathology following viral infection despite maintaining viral loads comparable to younger mice. These outcomes were associated with heightened systemic and tissue inflammatory cytokine production, reduced antigen-specific T cell responses, and increased frequencies of NK cells and non-antigen-specific bystander T cell activation. Coagulopathy with thrombolytic clot formation was observed exclusively in advanced aged mice. Transcriptomic analysis revealed enrichment of inflammatory and coagulation pathways in influenza-infected advanced aged mice, paralleling findings in elderly humans with SARS-CoV-2 infection, who also displayed reduced expression of T cell–associated genes.

**Discussion:**

These findings demonstrate that advanced age profoundly alters antiviral immune responses, shifting immunity away from effective antigen-specific T cell responses toward inflammatory and innate pathways that contribute to immune-mediated pathology. The results highlight the importance of modeling advanced aging in preclinical studies and suggest that age-dependent immune imbalance may underlie increased inflammation, coagulopathy, and mortality during viral infection in both mice and humans.

## Introduction

Aging is associated with a chronic inflammatory or “meta-inflammatory” state in a process known as “inflammaging” (1, 2) which is associated with the decreased ability to mount adaptive immune responses to new antigens. Chronic self-antigen stimulation through the release of damage associated molecular patterns (DAMPs) and chronic bacterial and viral infections releasing pathogen associated molecular patterns (PAMPs) become more difficult to resolve due to increased cellular senescence coupled with tissue deterioration, feeding into a cycle of chronic inflammation (1, 3). Age also contributes to the accumulation of adipose tissue, which is a major driver of pro-inflammatory cytokine production (1, 2, 4). Thymic involution occurs with aging as well, leading to a marked reduction in naïve T cell output and a peripheral T cell pool primarily consisting of memory T cells (5). As a consequence of thymic involution and inflammaging, aged immune responses are more dependent on memory or innate immune pathways against new antigen challenges (6). These processes put an aged individual at a higher risk of increased organ pathology following acute viral infections, which has been demonstrated clinically with numerous viral pathogens, including SARS-CoV-2 (7).

As the population shifts towards one that is predominantly elderly (8), it is apparent that preclinical modeling needs to accurately simulate the biological changes brought on with age. However, most studies investigating viral pathology *in vivo* utilize young animals, limiting extrapolation of results to the human scenario. Even within studies focusing on age, “age” in mice can be a broad term, with some using mice as young as 12 months of age as their aged cohort, and others using as high as 24+ months, offering some challenges when interpreting what is translatable due to mouse-to-human age equivalencies (per The Jackson Lab, this would be an age range of approximately 40–80 years old in humans) (9–19). Mice also have developed longer lifespans due to SPF housing conditions (20, 21), which has further complicated the definition of an “aged” mouse, with some strains now being able to survive 30+ months (9). Humans are defined as being “elderly” at 65 years of age (8), which would more closely correspond to a 20–24 month old mouse when accounting for prolonged longevity of SPF conditions (10). This may limit the translatability of studies that used younger mice to model aged pathology, as alterations in the immune profile that come with advanced aging could produce unique pathology in the context of viral infection or other immune challenges.

While studies have been performed in aged mice with both MCMV and influenza, results have been contradictory in some instances, with some suggesting aged mice have improved outcomes due to suppressed immunopathology (16), while others demonstrated the opposite, with aged mice having less efficacious adaptive immune responses and/or dysregulation of inflammation, experiencing more severe pathology (15, 18). The age of the aged cohorts was a variable in these studies, and this could partially explain the discrepancy in findings, warranting investigation of different ages of “aged” mice to determine translatability to human age equivalencies. This is also important in terms of designing immunotherapies, particularly in the scenario of unique pathology applying to age groups. An example of such unique pathology could be coagulopathy, as studies utilizing LPS, a potent toll receptor agonist, determined that dysregulation of coagulation mechanisms lead to mortality in the aged cohort (22).

Using multiple strains of mice, the pathology and immune responses to influenza and MCMV were investigated in mice delineated into young (2–6 months old), aged (15–18 months old), and advanced aged (20–29 months old) groups to determine if advanced age drove amplified pathology, or if unique pathology emerged, in comparison to young and aged mice. Infection with both MCMV and influenza revealed amplified immune pathology in advanced aged mice, with differentials manifesting between aged and advanced aged mice, showing a heightened degree of susceptibility to pathology with advanced age. Antigen specific T cell responses were reduced despite the aged recipients having similar viral titers compared to young recipients, while bystander T and NK cell responses were amplified. RNAseq revealed that elevated inflammatory pathways were observable between influenza infected advanced aged mice and SARS-CoV-2 infected 60–80 year-old humans, with genes associated with conventional T cell functionality being at lower expression in both species when compared to younger cohorts. Coagulopathy was found exclusively in the advanced aged cohort, suggesting that alterations in the advanced age immune profile can lead to unique pathology that must be accounted for when preclinically modeling age.

## Materials and methods

### Mice

C57BL/6 and BALB/c mice were purchased from The Jackson Laboratory (Bar Harbor, ME) and maintained until the appropriate age, being monitored for outwards abnormalities that would exclude them from the studies, such as the development of lesions or tumors. All mice were group-housed (up to 4 mice per cage) in AAALAC-approved specific pathogen–free facilities (The Institute of Regenerative Cures, University of California, Davis) with access to food and water (Teklad 2918 chow) and nesting for enrichment. The mice were housed in microisolator cages and experienced 12hr light/12hr dark cycles. Mice that were infected with MCMV were monitored at least twice per day and the studies adhered to Institutional Animal Care and Use Committee’s (IACUC) guideline of humane endpoints. Mice were euthanized when weight loss was greater than 20% and/or mice became moribund. Mice undergoing intranasal influenza infection were anesthetized using isoflurane vaporized in oxygen at 3–4% for induction and maintained at 1–2% during procedures. For terminal procedures, mice were euthanized using isoflurane overdose (≥5% isoflurane in oxygen, delivered in an induction chamber until respiratory arrest), followed by cervical dislocation, in accordance with UC Davis IACUC guidelines. All experimental protocols were approved by the IACUC of the University of California, Davis.

### MCMV stock and infection

The Smith strain of MCMV was originally obtained from American Type Culture Collection (ATCC) (Manassas, VA, BR-1399), maintained by repeated salivary gland passage in BALB/c mice, and quantified via plaque-forming assay with M2-10B4 cells (ATCC, Manassas, BA, CRL-1972), as described elsewhere (23). MCMV was administered intraperitoneally (i.p.) in 0.2 mL of RPMI 1640 medium (see figure legends for specific dose administered). Control mice were injected with 200 μL of RPMI 1640 i.p. Prior dose titrations were performed with young, healthy mice (both C57BL/6 and BALB/c) to determine dosages that were sublethal and lethal to their age range. Duration of studies and timepoints used to assay mice are described in figure schemas.

### Influenza stock and infection

Mice were anesthetized with isoflurane and infected intranasally with a sublethal to young mouse dose (10 PFU/ml) of influenza A/Puerto Rico/8/34 (A/PR8) (ATCC, Manassas, BA, VR-95) in 40 µl of PBS. The virus was grown in hen eggs as previously outlined (24), and each virus batch was titrated using young, healthy mice to determine sublethal and lethal doses for their age range. Duration of studies and timepoints used to assay mice are described in figure schemas.

### Processing of mouse tissues

Following euthanasia, organs were collected and placed into phosphate buffered saline (PBS) (Thermo Fisher Scientific, Waltham, MA, 14190094) on ice. Spleens and livers were mechanically homogenized into single cell suspensions for counting and flow cytometry staining, while a GentleMacs dissociator (Miltenyi Biotec, Bergisch Gladbach, North Rhine-Westphalia, Germany, 130-093-235) was used for the lungs. Liver cells were enriched by density centrifugation with 32% Percoll (Millipore Sigma, GE17-0891-02) in wash buffer (3% Nu-Serum in PBS). Cells were extracted from lung tissues using 0.1 mg/mL DNase I (Thermo Fisher Scientific, EN0521) and 1 mg/mL collagenase type IV (Millipore Sigma, C4-BIOC) in Hank’s Balanced Salt Solution (HBSS) (Thermo Fisher Scientific, 14170120), being incubated for 30 minutes at 37 degrees C after homogenization. The lung homogenate was further dissociated by being repeatedly drawn through an 18 G needle and syringe, and incubated for an additional 10 minutes at 37 degrees C. The spleen, liver, and lung suspensions were treated with red blood cell (RBC) lysis buffer (Thermo Fisher Scientific, 00-4333-57) for 5 minutes, washed with PBS, and then resuspended in PBS. Cells were counted using a Coulter Counter (Beckman Coulter Life Sciences, Brea, California) and then resuspended at 20 million cells per milliliter in staining buffer (3% fetal bovine serum in PBS).

Unless otherwise specified, immune phenotyping experiments were performed on single-cell suspensions prepared independently from lung, liver, and spleen tissues, and tissues were analyzed separately rather than directly compared across organs.

### Viral titer quantification

Single cell suspensions and tissue samples were snap-frozen in liquid nitrogen for MCMV viral titer quantification via real-time PCR. DNA was extracted from livers using the DNeasy Tissue Kit (Qiagen, Hilden, Germany, 69504) and the MCMV IE-1 gene was amplified using the HotStarTaq Master Mix (Qiagen, 203443) and forward and reverse primers. A standard curve was constructed by plotting cycle threshold (CT) value against log of IE-1-containing plasmid, followed by a sum of least square regression analysis. Plasmid was purified and quantified by serial 10-fold dilutions using forward and reverse primers and probe. Target copy numbers in the tissue samples were then calculated using the equation obtained by least squares regression analysis. Results were expressed as IE-1 gene copies/100 ng of DNA. Data was analyzed on CFX MaestroTM Software (Bio-Rad, Hercules, California).

### Flow cytometry

Cells were prepared in a single-cell suspension, washed with staining buffer, incubated with Fc block (BioLegend, San Diego, California, anti-CD16/32, clone 93, 101301), and then stained with the following fluorochrome-conjugated antibodies: CD45-PacBlue (BioLegend, clone 30-F11, 103125), CD45-FITC (Thermo Fisher Scientific, clone 30-F11, 11-0451-82), CD3-BV785 (Biolegend, clone 17A2, 100231), PD-1-PE/Cy7 (Biolegend, clone RMP1-30, 109109), TIGIT-APC (Biolegend, clone 1G9, 142105), NK1.1-PE-TR (Thermo Fisher Scientific, clone PK136, 12-5941-82), and CD69-FITC (BD Biosciences, clone H1.2F3, 553236). Live/dead staining was performed using Aqua (Thermo Fisher Scientific, L34957) or Zombie NIR Viability Dye (Biolegend, 423105). All intracellular staining was completed using the eBioscience Fixation/Permeabilization Solution Kit (Invitrogen, Waltham, MA, 88-8824-00). The stained cells were ran on a BD LSRFortessa flow cytometer (BD Biosciences, Franklin Lakes, New Jersey) and the data was analyzed using FlowJo v10 Software (BD Biosciences).

### Cytokine quantification

Serum cytokines were measured via BD Biosciences Mouse/Rat Soluble CBA Master Kit (BD Biosciences, 560232). Flex sets for IL-6 (BD Biosciences, 558301), TNF (BD Biosciences, 558299), IFN-γ (BD Biosciences, 558296), and IL-1β (BD Biosciences, 560232) were used with reagents from the master kit, according to the manufacturer’s instructions. Briefly, serum samples were diluted 1:4 with the included assay diluent and mixed with capture beads specific for the cytokines. PE detection reagent was added, and the samples were analyzed via flow cytometry using a BD LSRFortessa Flow Cytometer. The resulting MFI was used to calculate the cytokine levels in the serum as quantified in pg/mL based on the standard curve generated with the flex sets’ included standards. See figure legends for timepoint of blood collection for serum cytokine analysis.

### Histopathological assessment

Formalin-fixed tissues (lung and liver) were paraffin-embedded, then sectioned and stained with hematoxylin and eosin (H&E). A veterinary pathologist (E.C.) scored the tissues in a blinded manner. See the accompanying Supplementary figures for more information on the scoring metric used.

### RNA sequencing

Messenger RNA (mRNA) was isolated from total RNA using poly-T oligo-attached magnetic beads. This RNA was subsequently fragmented, and the first strand of complementary DNA (cDNA) was synthesized using random hexamer primers. This was followed by second-strand cDNA synthesis. The resulting library was evaluated using Qubit for quantification and real-time PCR, and a bioanalyzer was used for size distribution detection. Quantified libraries were then pooled and sequenced using a 2 × 150 bp paired-end method on Illumina NovaSeq 6000 sequencer. Human datasets were obtained through the Gene Expression Omnibus data repository (GSE161731) (25) or through the Genotype Tissue Expression Portal (https://gtexportal.org).

### RNAseq data analysis

The raw reads in fastq format were aligned to the mm10 reference mouse genome using Hisat2 (v2.0.5) [x1]. FeatureCounts (v1.5.0-p3) [x2] was employed to count the number of reads mapping to each gene. Differential gene expression analysis was carried out using the DESeq2 R package (v1.40.2) [23]. The P-values obtained from this analysis were adjusted using the Benjamini and Hochberg’s approach to control the false discovery rate. Genes with an adjusted P-value ≤ 0.05 and a fold change ≥2 were identified as differentially expressed. The “fgsea” R package (v.1.26.0) [24] was employed to conduct gene set enrichment analysis and to visualize the enriched gene sets. A clustered heatmap was generated using the regularized log-transformed data with the help of the “pheatmap” R package (v.1.0.12).

### Statistics

Statistical analysis was performed using GraphPad Prism v6.02 (GraphPad Software Inc., CA, USA). Data were expressed as mean ± standard error of the mean (SEM). A non-parametric Mann-Whitney U-test was used to compare two unpaired groups. For analysis of three or more groups, the non-parametric ANOVA was performed with a Bonferroni post-test. An adjusted p value of <0.05 was considered significant. Experiments were performed 2–4 times, as further specified in figure legends. Mouse numbers per group and per harvest ranged between 1–8 mice, with numbers shown in figures representing combined harvests through repeated experiments.

### Study approval

All animal studies and protocols were approved by the UC Davis IACUC (protocol numbers 18940 and 20707). Viral studies were approved by UC Davis biohazard use authorization (BUA) number R1592.

## Results

### Young, aged, and advanced aged mice and humans have distinct baseline T cell phenotype differences in different tissues

**Figure 1:**
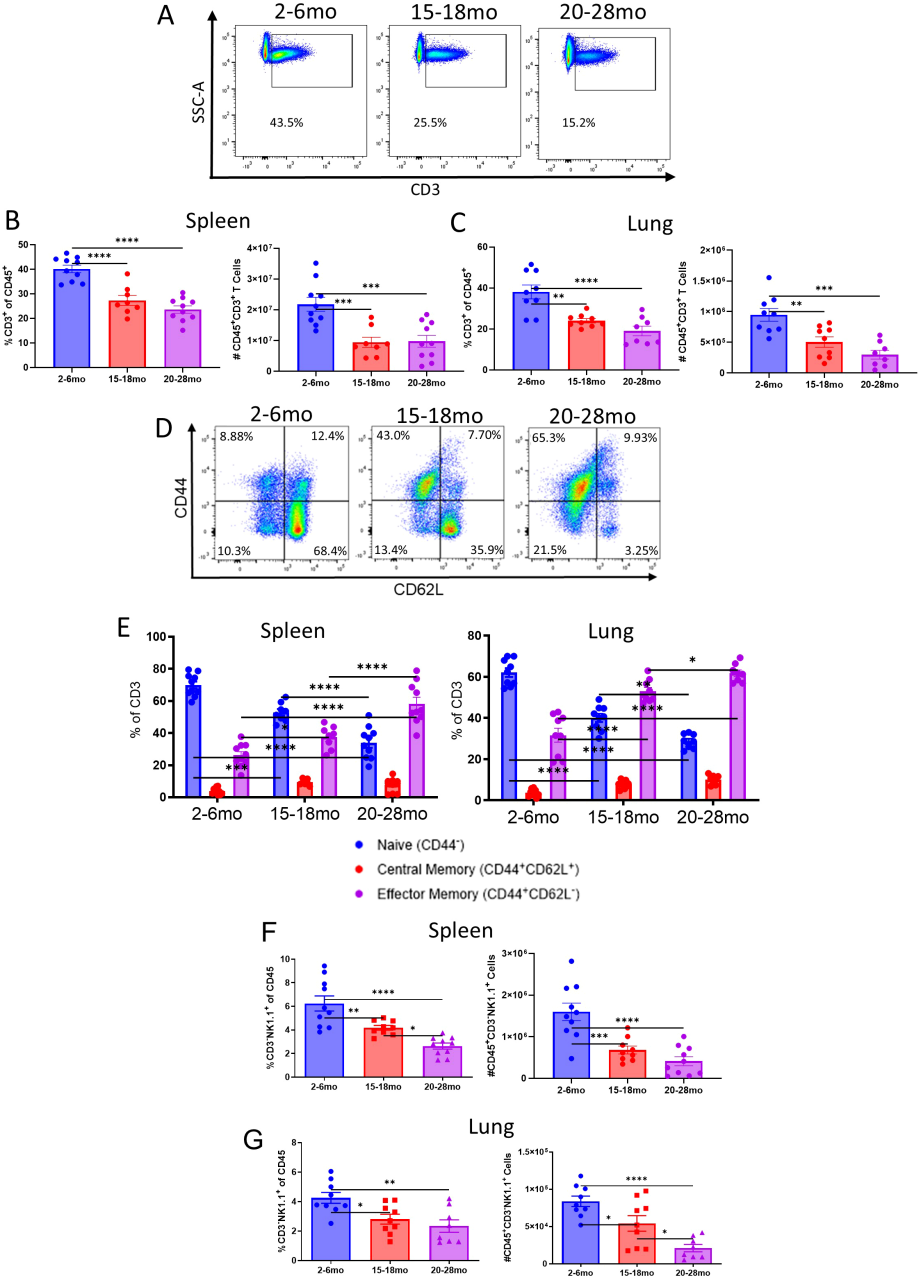
Young, aged, and advanced aged mice and humans have distinct baseline T and NK cell phenotype differences. **(A)** Representative flow cytometry staining of CD3 T Cells, as determined by gating CD45^+^NK1.1^-^ cells on CD3^+^, from the spleens of 2-6mo (young), 15-18mo (aged), and 20-28mo (advanced aged) mice with no prior treatment / infection. **(B, C)** Quantification of CD3^+^ cells previously gated on CD45 and cell counts for spleens (left) and lungs (right) of young, aged, and advanced aged mice. **(D)** Representative flow staining of young, aged, and advanced aged mice for naïve and memory T cell markers, previously gated on CD45^+^CD3^+^NK1.1^-^. **(E)** Breakdown of naïve (CD44^-^), central memory (CD44^+^CD62L^+^) and effector memory (CD44^+^CD62L^-^) CD3^+^ T cell populations in the spleens (left) and lungs (right) of young, aged, and advanced aged mice. **(F, G)** Percents of NK cells in the spleens and lungs of young, aged, and advanced aged mice. **(B, C, F, G)** SEM bars, n=8-10 mice per group, representative of 3-4 experiments. One-way ANOVA with Tukey’s multiple comparisons based on means between groups was used to determine statistical significance; P<0.05*, P<0.01**, P<0.001***, P<0.0001****. **(E)** SEM bars, n=8-9 mice per group, representative of 3 experiments. Two-way ANOVA with Tukey’s multiple comparisons based on means between groups used to determine significance; P<0.05*, P<0.01**, P<0.001***, P<0.0001****.

It has been observed previously that the aged environment contributes to phenomena such as T cell exhaustion and memory skewing, but it has not been established if there is a terminal point of T cell phenotype alteration that is reached upon a certain age. To determine this, the baseline T cell phenotype of 2–6-month-old (“young”), 15-18-month-old (“aged”), and 20+-month-old (“advanced aged”) mice were determined in key organs that would be the targets of acute viral infections, such as the lungs and liver, or otherwise representative of the peripheral immune system, like the spleen. Aging brought about a reduction in CD3^+^ T cells in the spleen and lungs, while advanced age drove these reductions further, particularly in the lungs (**Figures 1A–C**). An age dependent effect was also observed on memory T cell skewing, with the ratio of memory (CD62L^-^CD44^+^) to naïve (CD62L^+^CD44^-^) T cells increasing in aged and especially advanced aged mice (**Figures 1D, E**). T cell exhaustion, as determined through programmed cell death protein 1 (PD-1) staining, also gradually increased with age, again reaching a peak with advanced age (**Supplementary Figures 1A, B**). Age-associated increases in PD-1 expression on T cells have been widely reported in both murine and human studies and are linked to memory skewing, chronic antigen exposure, and exhaustion-like phenotypes that emerge with aging (26–28).

Baseline differentials in NK cells were also determined across the different ages. An age associated gradual decrease in NK cells, by both percentages and cell numbers, was observed in the spleens and lungs of aged and advanced aged mice (**Figures 1F, G**). An increase in TIGIT, a marker generally associated with NK cell “exhaustion” or impaired function, was also determined to gradually increase with age (**Supplementary Figures 1C, D**). Increased TIGIT expression on NK cells has been associated with impaired cytotoxicity and altered effector function in settings of chronic inflammation and cancer, though not explicitly with aging until now, consistent with an inhibitory or dysfunctional NK cell phenotype (29–33).

To determine if these age-related T and NK cell effects could be observed in the heterologous human population, RNA-seq data compiled nationwide, gathered from hundreds of patients of diverse backgrounds, were obtained from the Genotype Tissue Expression Portal (https://gtexportal.org). The data were stratified by age and analyzed for genes associated with T cell exhaustion, comparing groups aged 20-39, 40-59, and 60–79 years. RNAseq data from the blood of these patients revealed that several key genes associated with T and NK cell exhaustion were upregulated in older age groups, such as *Pdcd1*, *Lag3, Eomes, Ctla4, Tigit*, and *Havcr2*, among others (**Supplementary Figure 2A**). Similar observations were apparent in the lungs, with many T cell exhaustion genes analyzed being significantly elevated in older groups (**Supplementary Figure 2B**). In both, the blood and lungs, upregulation of these genes was most apparent in the 60–79 year old group, mirroring mouse data which demonstrated an age dependent effect on T cell exhaustion. These mouse and human data, when taken together, would indicate that the effects of aging on T cell phenotype occur gradually and become more pronounced with advanced aging.

### Advanced age is associated with organ driven pathology and mortality during acute MCMV infection

**Figure 2:**
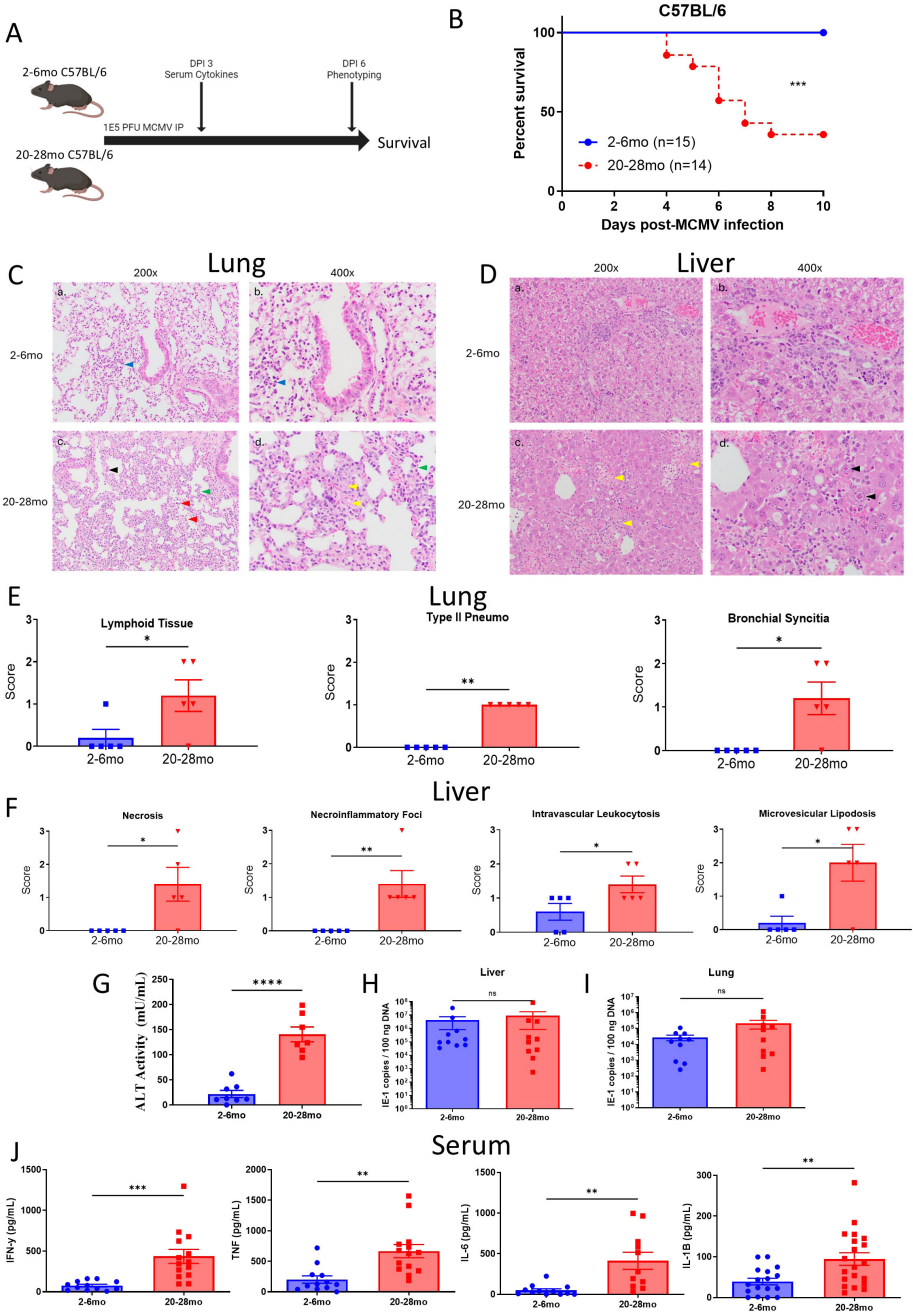
Advanced age is associated with organ driven pathology and mortality during acute MCMV infection. **(A)** Schema; C57BL/6 mice, aged between 2-7 months old (young) or 20-28 months old (advanced aged), were infected with 1E5 PFU MCMV intraperitoneally and assessed over the course of 10 days for acute viral infection survival. Blood was collected at DPI 3 for serum cytokines / ALT, and a cohort of the mice were assessed at day 6 for immune phenotype (mice used for this assessment were not included in survival data). Mice were euthanized based on moribund status / weight loss exceeding 20%. See **Supplementary Figure 2** for further information. **(B)** Young and advanced aged mouse survival curves after being infected with MCMV and euthanized at human endpoints. **(C)** Lung histology from 2-6 and 20-28mo mice at DPI6 of MCMV. (a, c) 200x views highlight the overall difference in interstitial inflammatory hypercellularity. In a) the interstitial hypercellularity is patchy and mild (blue arrowhead) whereas in c) it is widespread and moderate to marked (green arrowhead). (c) mild type II pneumoctes hyperplasia (black arrowhead) and vessel with increased intraluminal inflammatory cells with adherence to the endothelium (red arrowhead). (c, d) Higher magnification highlights thicker alveolar septa in the advanced aged mouse. Focus of fibrin exudation (yellow arrowhead) supports coagulopathy. **(D)** Liver histology from 2-6 and 20-28mo mice at DPI6 of MCMV. (a, b) Young mouse with mild, multifocal, lymphohistiocytic hepatitis without obvious necrosis (c, d) Advanced aged mouse with multifocal random necrosis and lymphohistiocytic inflammation (yellow arrowhead). Higher magnification in regions of necrosis and inflammation contain characteristic intranuclear inclusion bodies for MCMV infection (black arrow). **(E, F)**. Lung and liver H&E scoring of MCMV infected young and advanced aged mice at DPI6, as performed by a board certified pathologist. See **Supplementary Figure 2** for scoring metric. **(G)** Serum ALT levels of MCMV infected C57BL/6 young and advanced aged mice at DPI 6. **(H, I)**. Viral loads (IE-1 copies) in the organs of MCMV infected young and aged C57BL/6 mice at DPI 6, with liver **(H)** and lungs **(I)** on the right, as determined by PCR. **(J)** Serum levels of the inflammatory cytokines IFN-y, TNF, IL-6, and IL-1beta, taken from MCMV infected young and advanced aged C57BL/6 mice at DPI 3. **(B)** Survival analysis was plotted according to the Kaplan-Meier method, and statistical differences were determined with the log-rank test. P < 0.001***. **(E–J)** SEM bars, n=5 (histology scoring) or n=10 (viral loads and cytokines) mice per group, representative of 2-3 experiments. Two tailed student’s *t* test used to determine statistical significance; P<0.05*, P<0.01**, P<0.001***, P<0.0001****.

With baseline differentials established, it was next determined if advanced aged mice, which had the most signs of potential immune dysfunction, had altered outcomes in comparison to young mice when challenged with a systemic acute MCMV viral challenge. Young and advanced aged C57BL/6 and BALB/c mice were challenged with similar viral inoculums, previously determined to be minimally lethal in young mice (data not shown), to determine if survival and pathology differences could be observed (**Figure 2A**; **Supplementary Figure 2A**). In both mouse strains, advanced aged mice became rapidly moribund, with mortality occurring as early as day 4 and peaking between days 6–8 after infection, with ~50%+ mice not surviving the experiments (**Figure 2B**; **Supplementary Figure 3B**). Outwards pathology scoring and weight loss were also much more severe in advanced aged mice, which, corresponding to survival, began around day 4, and peaked around days 6–8 after infection (**Supplementary Figures 3C–G**).

Because pathology and mortality began to peak at day 6, liver and lung pathology was assessed at this timepoint via H&E and scored by a pathologist. The lungs of advanced aged C57BL/6 mice were significantly more impacted, showing elevated levels of lymphoid accumulation, type 2 pneumocyte hyperplasia, and branchial syncytia, which have been associated with worse organ pathology in the context of MCMV infection (**Figures 2C, E**). The livers of the advanced aged mice also had worse overall pathology, with necrosis, microvesicular lipidosis, intravascular leukocytosis, and necroinflammatory foci scoring significantly higher than what was observed in young mice (**Figures 2D, F**). This liver histology corresponded to elevated levels of ALT activity in the serum of advanced aged mice, a marker associated with liver damage (**Figure 2G**). At baseline, the organs of young and advanced aged mice showed no signs of the organ pathology that was assessed within the context of MCMV related damage (**Supplementary Figures 3H, I**).

It was next determined if this increased pathology and mortality was due to increased tissue viral loads. Surprisingly, the lungs and livers of the advanced aged C57BL/6 mice had comparable viral loads in comparison to their younger counterparts, despite having elevated organ pathology and mortality (**Figures 2H, I**). However, systemic levels of inflammatory serum cytokines (IFN-y, TNF IL-6, and IL-1B) (**Figure 2J**) and their associated genes in the lungs (**Supplementary Figure 3J**) were significantly elevated, creating a “cytokine storm.” These data would indicate that the mice were not succumbing to elevated viral pathology, but that the host immune response was amplified to the point of being deleterious.

### The dysregulated immune response of advanced aged mice is associated with diminished antigen-specific T cell responses but amplified bystander T and NK cell expansion/activation during acute viral challenge

**Figure 3:**
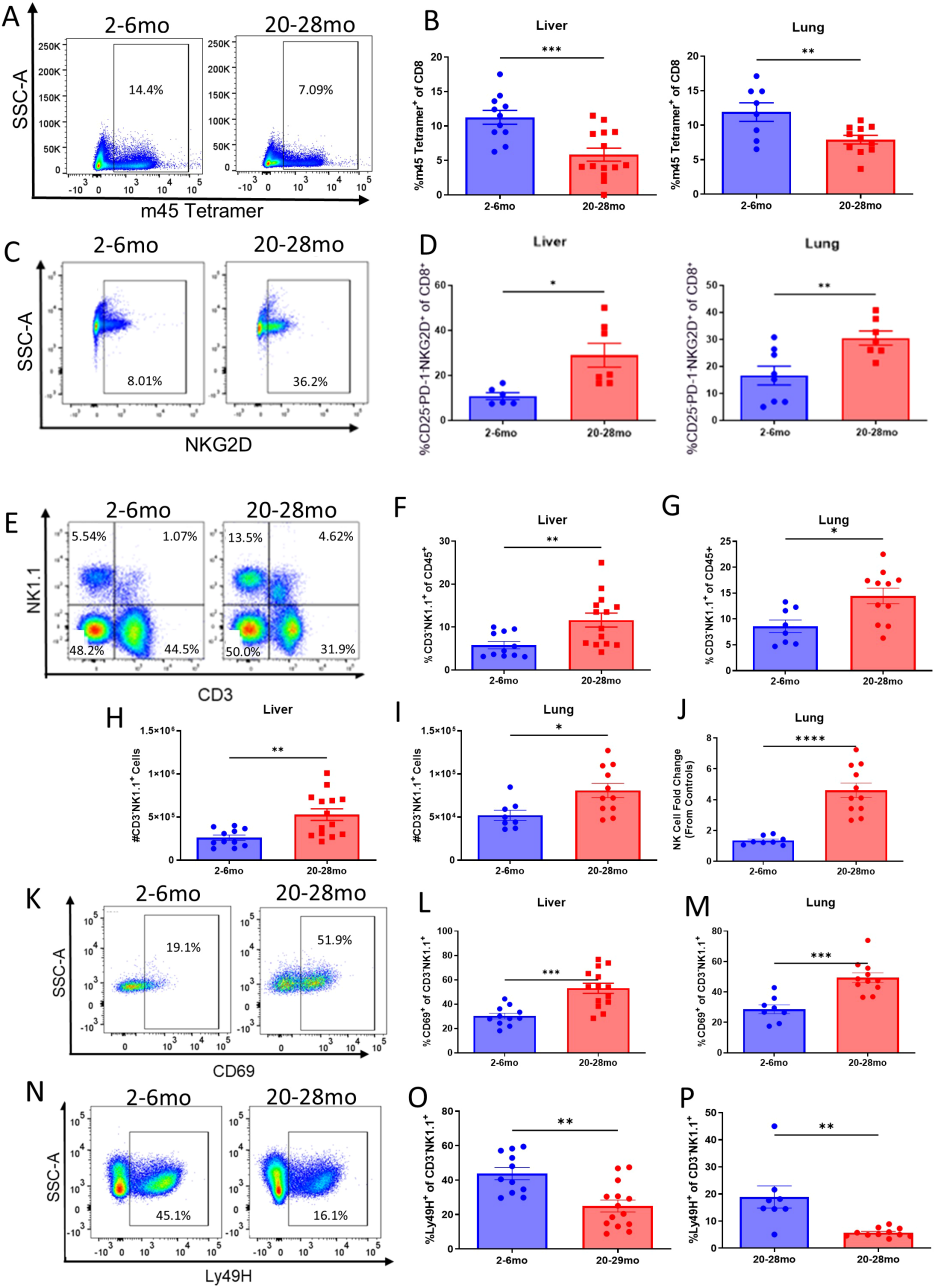
Advanced aged mice have an impaired antigen specific T cell response and amplified bystander T and NK cell expansion / activation during acute MCMV infection: Refer to **Figure 2A** for schema. **(A)** Representative flow staining of m45 tetramer in MCMV infected young and advanced aged C57BL/6 livers, taken at DPI 6. Parent gating: CD45^+^CD3^+^NK1.1^-^CD8^+^**(B)**. Percentages of tetramer positive CD8 T cells in the livers and lungs of MCMV infected young and advanced aged mice at DPI 6. **(C)** Representative flow staining of NKG2D for bystander T cell activation (same conditions / timepoint as **(A)**. Parent gating: CD45^+^CD3^+^NK1.1^-^CD8^+^CD25^-^PD-1^-^**(D)**. Percentages of bystander activated CD8 T cells in the livers and lungs of MCMV infected young and advanced aged mice at DPI 6. **(E)** Representative flow staining of NK cells (CD3^-^NK1.1^+^) in MCMV infected young and advanced aged C57BL/6 livers, taken at DPI 6. Parent gating: CD45^+^. **(F–I)** Percentages **(F, G)** and absolute counts **(H, I)** of NK cells in the livers and lungs of young and advanced aged mice at DPI 6. **(J)** Fold change of NK cell absolute counts in the lungs of MCMV infected young and advanced aged mice when compared to young mice at DPI 6. **(K)** Representative flow staining of activated NK cells in young vs advanced aged mice. Parent gating: CD45^+^CD3^-^NK1.1^+^CD69^+^. **(L, M)** Percentages of activated NK cells (CD69^+^) in the liver and lung. **(N)** Representative flow staining of Ly49H^+^ NK cells in young vs advanced aged mice. Parent gating: CD45^+^CD3NK1.1^+^Ly49H^+^. **(O, P)** Percentages of Ly49H^+^ NK cells in the liver and lung. **(B, D, F–J, L, M, O, P)** SEM bars, n=5-14 mice per group, representative of 2-4 experiments. Two tailed student’s *t* test used to determine statistical significance; P<0.05*, P<0.01**, P<0.001***.

Adaptive T cell responses to viral challenge were next assessed in the two cohorts. The induction of MCMV-specific response by T cells was determined by using m45 tetramer, marking antigen-specific CD8 T cells against MCMV. Advanced aged mice had significantly lower levels of tetramer-positive T cells in the liver and lung when compared to their younger counterparts, indicative of an impaired antigen specific T cell response (**Figures 3A, B**). Bystander T cell activation, in which memory T cells become activated in a non-antigen specific manner in high cytokine/inflammatory environments and mediate effector cell functions via NKG2D receptor and not TCR binding, was then assessed. It was determined that bystander T cells were significantly elevated in advanced aged mice as determined by presence of activated T cells not expressing CD25 or PD-1, which occurs after TCR crosslink-linking (**Figures 3C, D**; bystander phenotype CD25^-^PD-1^-^NKG2D^+^ and/or CD25^-^PD-1^-^CD69^+^) (34–36).

A significant increase in the expansion of NK cells was observed in the livers and lungs of the infected advanced aged mice, indicative of compensatory innate immune pathways (**Figures 3E–I**). The lungs in particular had a marked fold increase in NK cell numbers, which was of particular note due to them having lower amounts of NK cells at baseline (**Figures 1G**, **3J**). While the NK cells of advanced aged mice were found to have significantly higher expression of CD69, an activation marker (**Figures 3K–M**), levels of Ly49H, an activating NK cell marker that specifically recognizes the m157 ligand of MCMV infected cells, were significantly lower (**Figures 3N–P**). These data would indicate that, in potential compensation for an impaired antigen specific response against the virus, innate pathways by NK cells are amplified.

**Figure 4:**
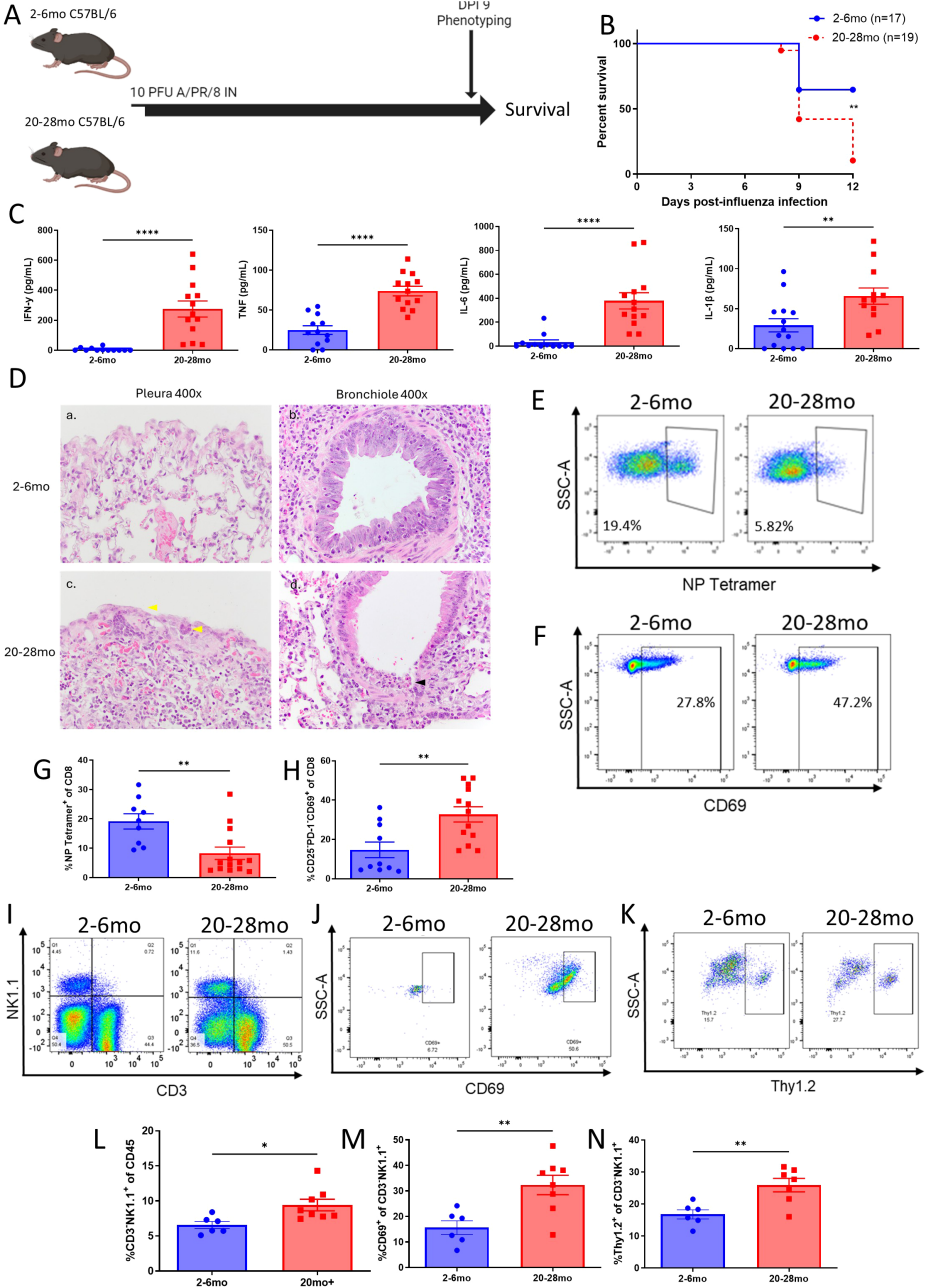
Influenza immune mediated pathology is amplified in advanced aged mice. **(A)** Schema: 2-7mo (young) and 20-28mo (advanced aged) mice were infected intranasally with 10 PFU of A/PR/8 influenza and immune phenotyped at DPI9. Mice used for assessment were not included in survival data. **(B)** Survival curve of influenza infected mice during acute phase of influenza infection with humane endpoints. **(C)** Lung supernatant levels of IFN-y, TNF, IL-6, and IL-1B, as determined by CBA. **(D)** Representative histology of influenza infected mouse lungs at DPI 9. (a) Relatively normal pleura and lung parenchyma from young mouse. (c) Advanced aged mouse with mesothelial hypertrophy (yellow arrow) and inflammation associated with inflammation of the parenchyma. (b) In young mice, intraepithelial lymphocytes associated with bronchiolar epithelial hyperplasia was common, in contrast to (d) segmental to diffuse bronchiolar epithelial necrosis seen in advanced aged mice. **(E, F)** Representative staining of NP tetramer **(E)** and bystander activated CD69^+^**(F)** CD8^+^ T cells in the infected lungs of young and advanced aged mice. Previous gating: CD45^+^CD3^+^NK1.1^-^CD8^+^ (tetramer), CD45^+^CD3^+^NK1.1^-^CD8^+^CD25^-^PD-1^-^ (bystander). **(G, H)** Percentages of tetramer positive **(G)** and bystander activated CD69^+^**(H)** CD8 T cells in the lungs of infected mice at DPI 9. **(I)** Representative staining of NK cells (CD3^-^NK1.1^+^), as well as activation on NK cells via CD69 **(J)** and Thy1.2 **(K)** staining, in the lungs of infected young and advanced aged mice. Parent gating: CD45^+^ [for **(I)**], CD45^+^CD3^-^NK1.1^+^**(J, K)**. **(L–N)** Percents of NK cells **(L)** and activated NK cells **(M, N)** in the lungs of infected young and advanced aged mice **(B)**. Survival analysis was plotted according to the Kaplan-Meier method, and statistical differences were determined with the log-rank test. P < 0.01** **(C, G, H, L, M, N)**. SEM bars, n=7-10 mice per group, representative of 2 combined experiments. Two tailed student’s *t* test used to determine statistical significance; P<0.05*, P<0.01**; P<0.001***, P<0.001***.

It was next determined if influenza, a more respiratory centric virus, could yield similar differentials in pathology and immune phenotype in young and advanced aged mice (**Figure 4A**). While young mice demonstrated outwards pathology and mortality at the dose used, advanced aged mice experienced significantly greater pathology in comparison and had a higher rate of mortality (**Figure 4B**; **Supplementary Figures 4A, G**). Lung inflammatory cytokines levels and cytokine gene expression (IFN-y, TNF, IL-6, and IL-1B) were significantly elevated in comparison to younger mice (**Figure 4C**; **Supplementary Figure 4C**) at the peak of the primary response (day 9), mirroring the systemically elevated levels found after MCMV infection. Lung pathology, as determined by H&E staining, was also markedly increased in advanced aged mice (**Figure 4D**), with necrosis, bronchiolar necrosis, pleural involvement, and intraepithelial leukocyte scores being significantly higher (**Supplementary Figures 4B, J**). Similarly to what was observed with MCMV infection, the frequencies of antigen specific CD8 T cells to influenza, measured by NP tetramer, were significantly lower in advanced aged mice lungs compared to young adult mice on day 9 of infection (**Figures 4E, G, I**, **Supplementary Figures 4E, G**). Bystander T cell activation was also similarly significantly higher in advanced aged lungs (**Figures 4F, H**). Evidence of coagulopathy in the form of luminal fibrin was found in the lungs of advance aged mice, while no coagulopathy was found in young lungs (**Supplementary Figure 4I**).

A significant increase in NK cells was observed in the lungs of the advanced aged mice in comparison to the young mice (**Figures 4J, K, M, N**). Reinforcing observations made from MCMV infection, these NK cells were found to express significantly higher amounts of activation associated markers, such as CD69 and Thy1.2 (**Figures 4I–N**). These data, taken in combination with observations made with MCMV infection, indicate that the advanced aged immune response lacks efficiency in mounting antigen specific T cell responses following acute viral infections, while non-antigen specific mechanisms are amplified, correlating with potentially immune mediated organ pathology.

### Advanced aged mice have further impaired adaptive T cell responses and elevated inflammatory cytokine production when compared to aged mice

**Figure 5:**
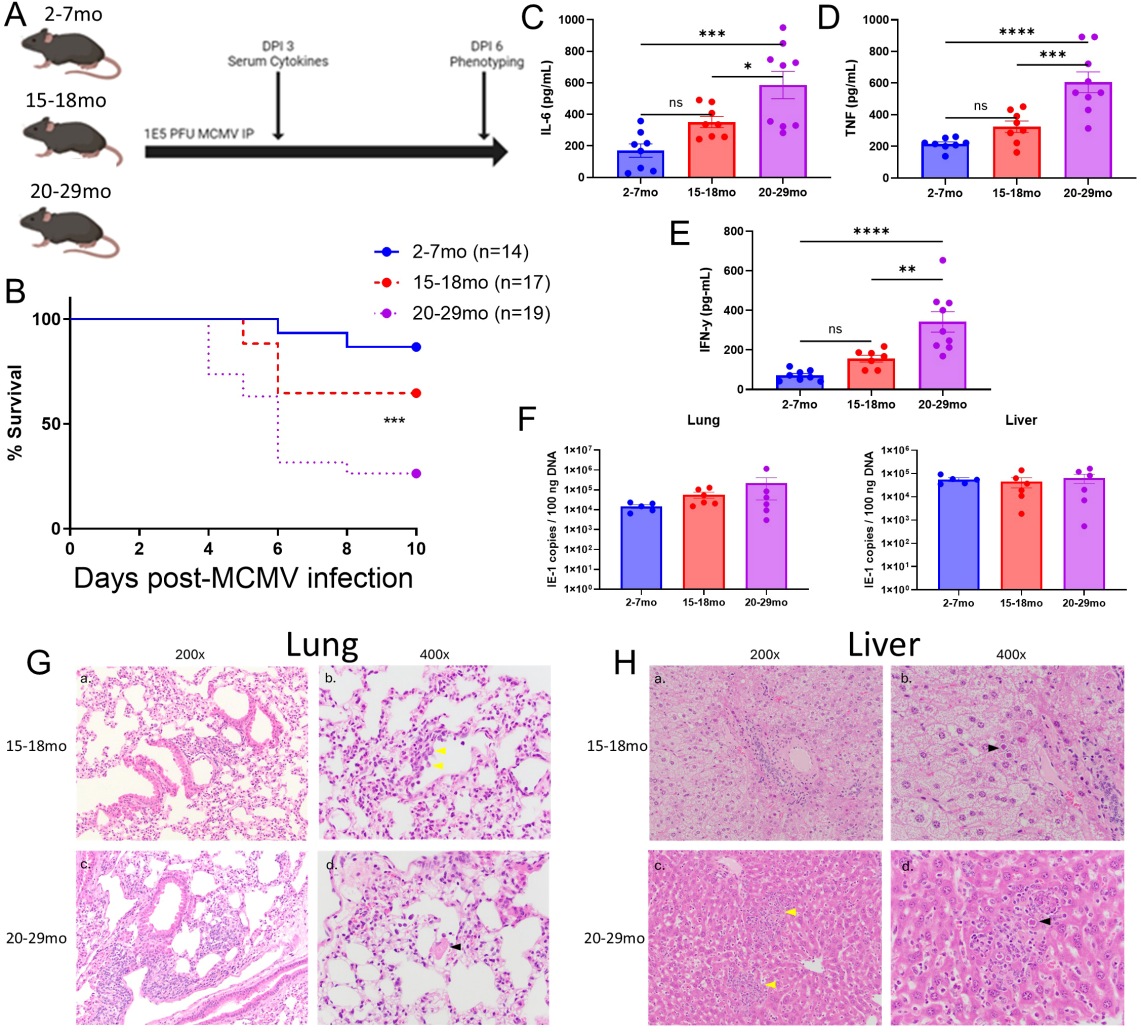
Age related acute viral pathology intensifies with further aging. **(A)** Schema; C57BL/6 mice, aged between 7, 15-17, and 24-29 months old, were infected with 1E5 PFU MCMV intraperitoneally and assessed over the course of 10 days for acute viral infection survival. Blood was collected at DPI 3 for serum cytokines, and a cohort of the mice were assessed at day 6 for immune phenotype (mice used for this assessment were not included in survival data). Mice were euthanized at humane endpoints based on moribund status / weight loss exceeding 20%. **(B)** Survival curves of mice after MCMV infection. **(C–E)** Serum levels of inflammatory cytokines IL-6, TNF, and IFN-y, DPI3 of MCMV infection. **(F)** Viral titers (IE-1 copies) of MCMV in the livers and lungs of infected mice at DPI. **(G)** Representative histology of 15-17 and 24-29mo mouse lungs at DPI6 of MCMV infection (a, b) 15-17mo mouse with mild, multifocal, lymphohistiocytic interstitial pneumonia with intravascular inflammatory cells yellow arrow) (c, d) 24-29mo mouse with moderate, multifocal interstitial pneumonia with rare intraalveolar fibrin exudation (black arrowhead). **(H)** Representative histology of 15-17 and 24-29mo mouse livers at DPI6 of MCMV infection. (a, b) 15-17mo liver with diffuse microvesicular lipidosis and intranuclear MCMV inclusion bodies (black arrowhead) without associated necrosis and inflammation. (c, d) 24-29mo liver with moderate, multifocal random hepatitis (yellow arrowhead) with similar inclusion bodies. **(B)** Survival analysis was plotted according to the Kaplan-Meier method, and statistical differences were determined with the log-rank test. P < 0.05*. **(C–E, F)** SEM bars, n=8-12 mice **(C-E)** or 7-8 mice **(F)**, representative of 2-3 experiments. One-way ANOVA with Tukey’s multiple comparisons based on means between groups was used to determine statistical significance; P<0.05*, P<0.01**, P<0.001***, P<0.0001****.

While efforts were focused on defining the differentials between young and advanced aged mice due to observations made at baseline indicating the that advanced aged mice had more potential for immune dysregulation (**Figure 1**; **Supplementary Figure 1**), a direct comparison between young, aged, and advanced aged mice was next performed next to assess whether viral immune response alterations in age progress over time (**Figure 5A**). While aged mice did suffer higher infection-induced mortality than young mice, advanced aged mice still had significantly higher mortality in comparison (**Figure 5B**). Serum cytokine levels of IL-6, TNF, and IFN-y were also notably elevated in what appears to be a gradual increase with age (**Figures 5C–E**), while viral control was comparable across age groups (**Figure 5F**). Critically, coagulopathy was again found in advanced aged mice target organs (lungs and liver), but not in aged mice, indicating that age incurred unique pathology distinct from that of other age groups, in addition to other indicators of immune dysregulation (**Figures 5G, H**). While this data does indicate that age gradually amplifies inflammatory responses and organ pathology while not offering benefits to viral control, it also suggests that unique pathology can manifest with advanced age that could be potentially missed by using younger mice, validating the distinction between “aged” and “advanced aged” mice.

**Figure 6:**
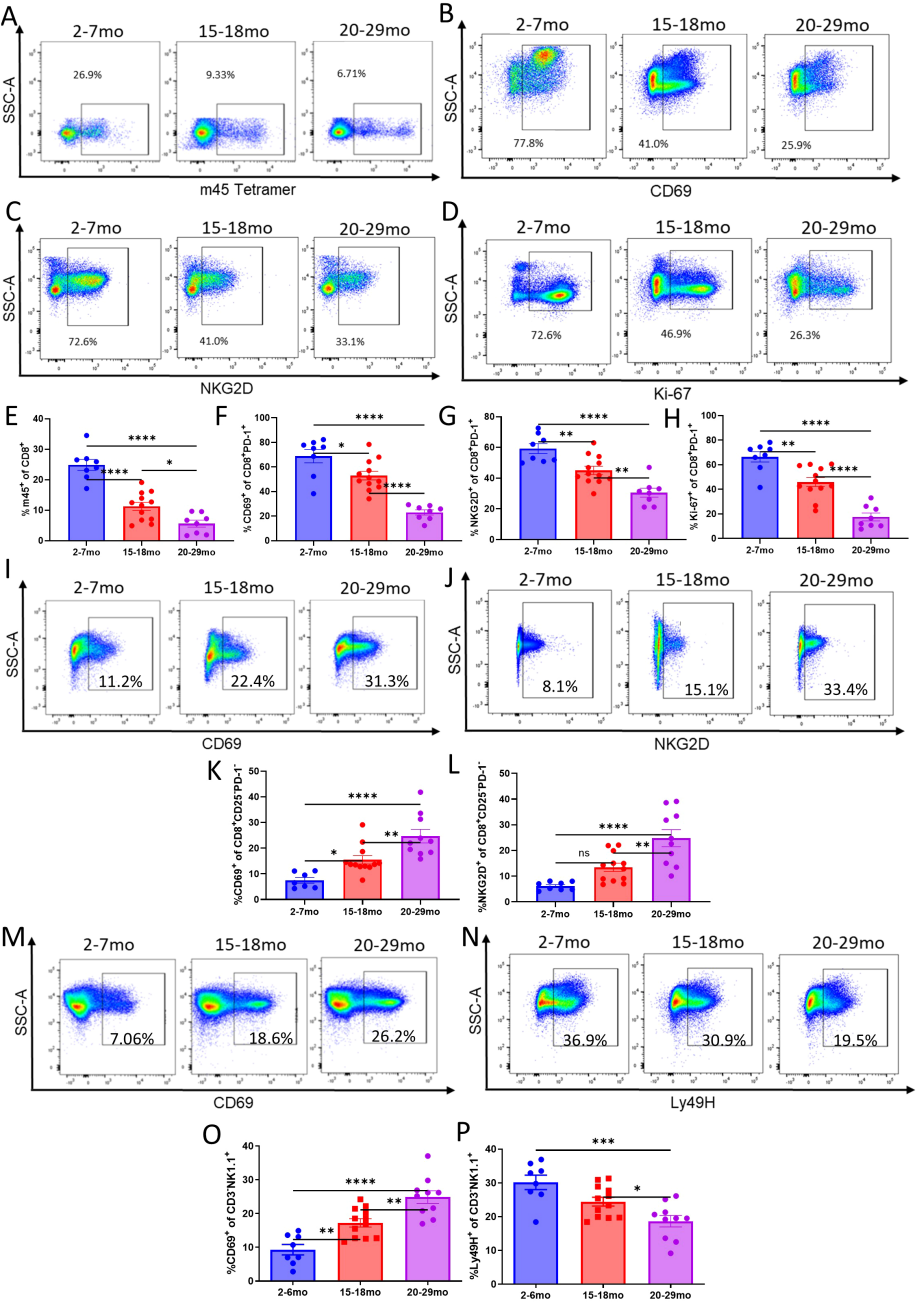
Immune dysfunction is gradually amplified with aging in the context of acute viral infection. **(A–D)** Representative flow staining of m45 tetramer (previously gated on CD45^+^CD3^+^NK1.1^-^CD8^+^), CD69, Ki-67, and NKG2D **(B–D)** previous gating - CD45^+^CD3^+^NK1.1^-^CD8^+^PD-1^+^) in the spleens of young, aged, and advanced aged mice assessed at DPI 6 of MCMV infection. **(E–H)** Percentages of tetramer positive CD8 T cells **(E)** and marker expression of CD69 **(F)**, NKG2D **(G)**, and Ki-67 **(H)** in the spleens of young, aged, and advanced aged mice assessed at DPI 6 of MCMV. Note that **(H–J)** were gated on PD-1 previously due to its upregulation associated with conventional T cell activation. **(I, J)** Representative flow staining of bystander activation via CD69 and NKG2D (Parent gating: CD45^+^CD3^+^NK1.1CD8^+^CD25^-^PD-1^-^) in the spleens of young, aged, and advanced aged mice assessed at DPI 6 of MCMV. **(K, L)** Percentages of bystander activated CD8 T cells in the spleens of young, aged, and advanced aged mice at day 6 DPI of MCMV. **(M, N)** Representative staining of activated (CD69^+^) and Ly49H^+^ NK cells in the spleens of young, aged, and advanced aged mice at DPI 6 of MCMV. (Parent gating: CD45^+^CD3NK1.1^+^). **(O, P)** Percentages of activated **(O)** and Ly49H^+^**(P)** NK cells in the spleens of young, aged, and advanced aged mice at DPI 6 of MCMV. **(E–H, K–L, O–P)** SEM bars, n=8-12 mice, representative of 2-3 experiments. One-way ANOVA with Tukey’s multiple comparisons based on means between groups was used to determine statistical significance; P<0.05*, P<0.01**, P<0.001***, P<0.0001****.

Antigen-specific CD8 T cell responses were next assessed using m45 tetramer, demonstrating similar significantly reduced frequencies of T cell responses, with those in advanced aged mice even more strongly reduced than those in the aged group when compared to the young in the spleens (**Figures 6A, E**). Markers of activation and proliferation, such as CD69, Ki-67, and NKG2D, were also lower in advanced aged animals, further demonstrating impairment in the T cell response (**Figures 6B–D, F–H**). Bystander activation was significantly higher in advanced aged mice vs aged mice, again signifying gradual age mediated amplification of the non-antigen specific response (**Figures 6I–L**).

NK cells were next assessed to determine if functionality was further altered with advanced aging. The NK cells of advanced aged mice demonstrated significantly higher activation via CD69 (**Figures 6M, O**). Ly49H was again lower in advanced aged mice, however (**Figures 6N, P**). It is notable that across the markers assessed, differentials between aged and advanced age mice were consistently discovered regarding NK and T cell phenotyping, offering evidence of significant differences between the age groups that should be considered when modeling the aged condition, and especially when attempting to translate data or test immunotherapies.

### Advanced aged drives dysregulation of inflammatory and coagulation pathways during acute viral infection in mice and humans

**Figure 7:**
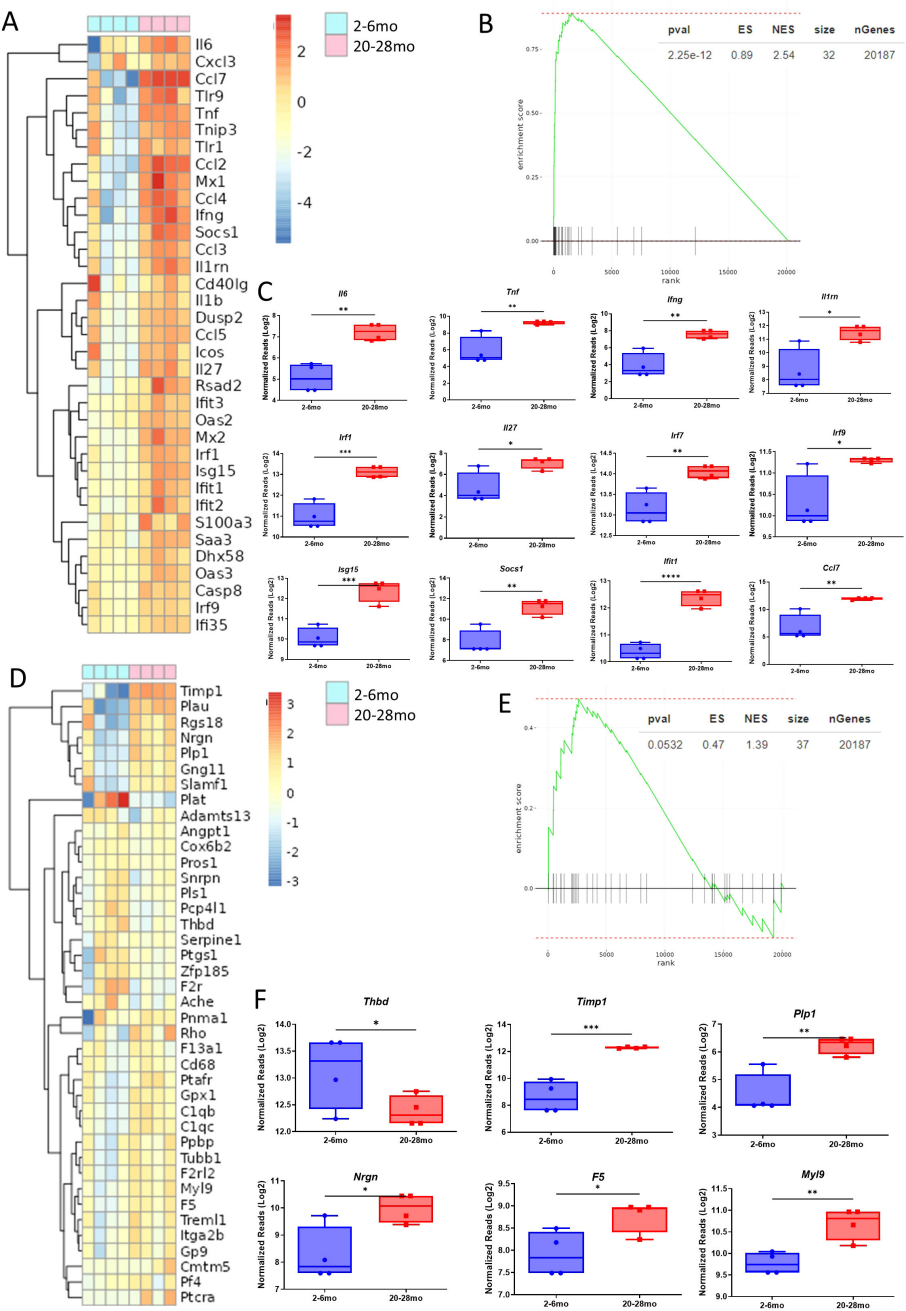
Advanced aged mice have dysregulated inflammatory and coagulative mechanisms during influenza infection. Schema of **Figure 4A** is applicable to this figure. **(A)** RNAseq heatmap of inflammation associated genes and their differential expression from the extracted RNA of lung cells from young and advanced aged influenza infected (DPI 9) mice. **(B)** GSEA of inflammation related genes in the heatmap of 7A, with advanced aged vs young mice as the basis of enrichment **(C)** Normalized reads of critical inflammatory genes in young and advanced aged infected mice. Data underwent a Log2 transformation, as well as Winsorization. **(D)** RNAseq heatmap of coagulation regulation associated genes and their differential expression from the extracted RNA of lung cells from young and advanced aged influenza infected (DPI 9) mice. **(E)** GSEA of coagulation related genes in the heatmap of 7D, with advanced aged vs young mice as the basis of enrichment **(F)** Normalized reads of critical coagulation genes in young and advanced aged infected mice. Data underwent a Log2 transformation, as well as Winsorization. **(C, F)**. SEM bars, n=4 mice per group, representative of 2 experiments. Student’s *t* test used to determine statistical significance; P<0.05*, P<0.01**, P<0.001***, P<0.0001****.

The lungs of young and advanced aged MCMV-infected mice were next compared via bulk RNAseq analysis to determine the underlying mechanisms guiding the dysregulated immune response. 2575 genes were shown to be differentially expressed between these groups, of which 1224 genes were upregulated, and 1351 genes downregulated (**Supplementary Figure 5B**). A heatmap of key inflammatory genes, such as *Il6, Tnf, Il1rn, Ccl7, Ifng*, and numerous others showed significant upregulation in advanced aged mice, evidence that supported previous observations of inflammatory dysregulation (**Figures 7A–C**; **Supplementary Figures 5A–D**). Genes associated with T cell exhaustion/impaired function/suppression were also found to be upregulated in advanced aged mice (*Pdcd1, Eomes, Lag3, Ctla4, Cd274*, and others*)*, while those associated with activation/proliferation were at lower expression (*Nfat1, Nfat3, Rorc, Zbtb16, Trem1*, and others*)* (**Supplementary Figure 5E**). Gene set enrichment analysis showed pathways associated with the positive regulation of IFN-β production, type 1 interferon signaling pathway, and defense response to viral infection were also upregulated in advanced aged mice compared to the young group (**Supplementary Figure 5F**).

It was next determined if dysregulated coagulation may be occurring, especially after complete blood analysis revealed elevated peripheral platelets during infection in advanced aged mice (**Supplementary Figure 5G**). Genes associated with the regulation of coagulation, such as *Thbd, Timp1, F5, Plp1*, and others were differentially expressed in advanced aged mice, with *Thbd* in particular having been shown by others to be particularly deleterious in the context of coagulopathy (22) (**Figures 7D–F**). With previous H&E staining showing evidence of coagulopathy unique to advanced aged mice with influenza, this data would support that the regulation of coagulation is altered with age, potentially allowing for coagulopathy to emerge during viral infection.

### Advanced human aging amplifies inflammatory mechanisms and alters coagulation pathways during SARS-CoV-2 infection

**Figure 8:**
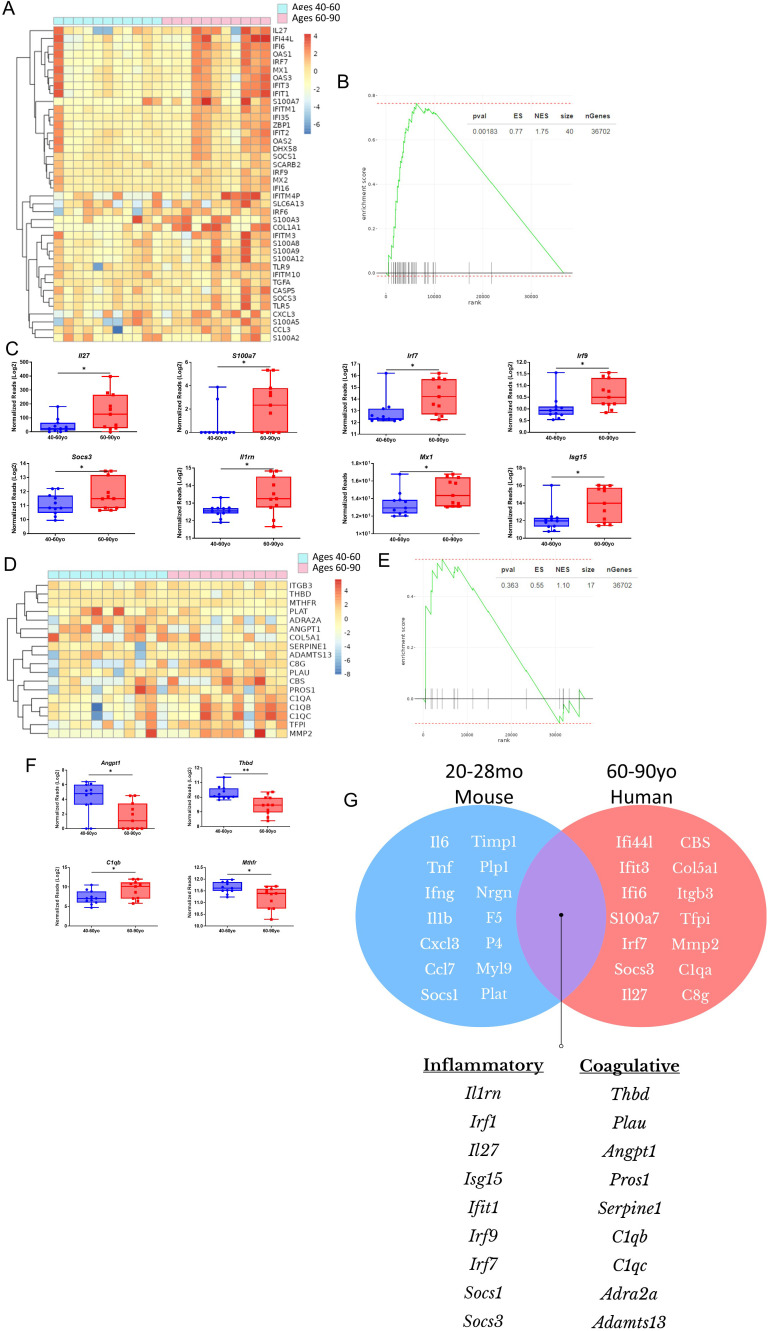
Advanced human aging amplifies inflammatory pathways and coagulative dysregulation during SARS-CoV-2 infection. **(A)** RNAseq heatmap of inflammation associated genes and their differential expression from the blood of SARS-CoV-2 patients, aged 40-59 and 60-79 years. **(B)** GSEA of inflammation related genes in the heatmap of 8A, with 60-79 vs 40-59 year olds as the basis of enrichment **(C)** Normalized reads of critical inflammatory genes shown in the heatmap of 8A. Data underwent a Log2 transformation, as well as Winsorization. **(D)** RNAseq heatmap of coagulation regulation associated genes and their differential expression from the blood of SARS-CoV-2 patients, aged 40-59 and 60-79 years **(E)**. GSEA of coagulation related genes in the heatmap of 8D, with 60-79 vs 40-59 year olds as the basis of enrichment **(F)** Normalized reads of critical coagulation genes in 7D. Data underwent a Log2 transformation, as well as Winsorization. **(G)** Venn-diagram graphical representation of coagulative and inflammatory genes found to be differentially expressed in advanced aged mice and 60-79 year old humans with infection when compared to younger groups. Genes overlapping (purple portion of diagram) were found to be differentially expressed in both species, while those not overlapping (blue for mouse, red for human) were found to be differentially expressed but in both species. It should be noted all inflammatory genes shown were upregulated, while coagulative genes were up- and down- regulated in comparison to younger groups. **(C, F).** SEM bars, n=8 patients per group. Student’s *t* test used to determine statistical significance; P<0.01**.

With evidence suggesting advanced aging exacerbates immune mediated pathology during acute viral infection, it was next determined if similar processes occur in human patients in the context of SARS-CoV-2 infection. RNAseq data gathered from the blood of infected SARS-CoV-2 patients (Gene Expression Omnibus data respiratory – dataset GSE161731) (25) was stratified based on age into groups of 40–60 vs 60–80 year old groups to determine if correlations could be made to that which was observed in mouse modeling. Between these groups, 711 genes were differentially expressed, of which 372 were upregulated, and 339 downregulated (**Supplementary Figure 6A**). A multitude of inflammatory genes were increased in the 60–80 year old group compared the younger aged group (**Figures 8A, B**), with genes like *Irf7, Irf9, Il1rn, Il27, and Socs3* being significantly elevated in the older group (**Figure 8C**). Analysis of genes associated with T cell activation and functionality, such as multiple *MAPK* genes (*Mapk11, Map3k11, Map2k2), Irak4, Nlrc4*, and numerous others, were expressed at lower levels in the older group (**Supplementary Figures 6B, C**). These data are thus similar to observations made in infected advanced aged mice.

Because altered coagulation pathways were observed in advanced aged mice during viral challenge, and because coagulopathy has been associated with SARS-CoV-2 morbidity and mortality, genes associated with regulation of coagulation were next compared between 40–60 and 60–80 year olds with SARS-CoV-2 infection. Again mirroring the data from mice, the group of older age humans had significant differentials in coagulation genes, with *Thbd* being at a markedly lower expression, but a multitude of other regulatory genes were also at altered expression (**Figures 8D–F**).

The inflammatory and coagulation profiles established in 60–80 year old humans corresponded to data from advanced aged mice. Inflammatory markers, such as *Il1rn, Irf1, Il27, Isg15, Ifit1, Irf9, Irf7, Socs1*, and *Socs3* were upregulated in infected older groups compared to the younger groups, providing evidence of age amplified inflammatory dysfunction (**Figure 8G**). Similarly, regulatory genes for coagulation, most notably *Thbd* but also others like *Plau, Angpt1, Pros1, Serpine1, C1qb, C1qc, Adra2a*, and *Adamts13*, were differentially expressed in the aged groups compared to the younger ones, indicating a component of aging in altered coagulation regulation (**Figure 8G**). These data offer substantive evidence that aging causes amplified inflammatory cytokine production, diminished antigen specific CD8 T cell responses, and alterations in coagulation.

## Discussion

Aging has been generally associated with poorer clinical outcomes in the context of viral infections as recently evidenced in the SARS-CoV-2 pandemic, which disproportionately affected the aged population (7). The immune system also undergoes significant alterations with age, with profound changes in T and NK cell numbers and phenotyping occurring. Despite this, most preclinical viral modeling examining pathogenesis and therapeutic interventions use young, inbred SPF laboratory mice, and even those that do use aged mice employ such a large range across studies that extrapolating can be an issue, and indeed, contrasting data has arisen concerning the aged condition. The data in this manuscript provides evidence for the need to perform age-equivalent preclinical studies, as demonstrated by observations of advanced aged mice exhibiting profound suppressed adaptive T cell responses, marked inflammatory-mediated pathology and mortality, and amplification of non-antigen specific mechanisms following acute viral challenge in two different viral models. Most importantly, these deleterious outcomes intensified with age and occurred despite comparable viral resistance, in which the impaired adaptive T cell responses of advanced aged mice were offset by significantly elevated bystander T cell activation and marked increases in activated NK cells in the infected tissues, suggesting innate immune responses compensated for the deficit in antigen-specific responses. Ly49H, a NK cell marker that recognizes MCMV, was significantly lower in advanced aged mice however, potentially evidencing that the NK cells, despite being activated, were still impaired in terms of actual response to the virus. Evidence of these altered transcriptional pathways were observed in both aged mice and humans following viral infection, demonstrating a common linkage between the species regarding the immune and inflammatory effects.

The data provided in this manuscript provide targets for immunotherapy in the context of aged and advanced aged viral infection, such as SARS-CoV-2. Clinically relevant immunotherapies, such as anti-IL-6 and anti-TNF, which have already seen limited application in human SARS-CoV-2 cases (37, 38), could be further investigated preclinically and clinically due to observations of cytokine storm and inflammatory dysregulation in this manuscript. These interventions could also be investigated for potential synergistic effects that have been noted in other models of overamplified cytokine production, like acute GVHD (39), though a balance must be achieved between immune regulation and maintaining an adequate immune response.

Because of observed elevated T cell exhaustion profiles at baseline with age and impaired T cell responses during infection, immune checkpoint blockade therapy could be a potential option in restoring T cell functionality, although the profound memory T cell skewing that occurs with age due to thymic involution may limit potential benefits. It is also worth considering that, even though the antigen specific T cell response was impaired in aged and advanced aged mice, that it may still be efficacious enough to overcome the virus, and that dysregulated innate mechanisms were interfering with T cell activity, which could be the case per evidence suggesting that NK cells act as rheostats against CD8 T cells during viral infection (40, 41). The marked increase in NK cells may also contribute to the overall augmented innate inflammatory pathways observed, and it’s worth noting that several NK cell associated genes, such as *Nkg7*, *Gzmb*, and *Prf1* were upregulated in humans and mice. Given that TIGIT was observed to gradually increase with age as well in NK cells, anti-TIGIT therapy could be an option in restoring NK cell functionality as well (30). Restoration of functionality may be difficult to conceptualize as the data in this manuscripts showed higher expansion and activation status with infection in NK cells, but importantly, Ly49H levels were actually lower in NK cells, suggesting impairment of NK cell mediated activity against MCMV (42), though an explicit correlation between TIGIT and Ly49H has not yet been determined. There is an inherent difficulty in delineating the positive and negative aspects of a particular immune cell-type in this context since cell depletion studies (ie for NK cells) or immunodeficient mouse models would result in greater susceptibility in general to viral infection and pathogenesis, even if it did protect from immune-mediated pathology.

Transcriptional profiling of infected lungs from advanced-aged mice revealed a coordinated immune shift characterized by reduced expression of activation and proliferation-associated genes, including NFAT1/3, RORγt, Zbtb16, and Trem1, alongside enrichment of antiviral and type I interferon signaling pathways. Reduced expression of these transcriptional regulators is consistent with impaired adaptive immune coordination and diminished antigen-specific T cell responses with advanced age, while sustained type I interferon signaling indicates persistent innate immune activation despite effective viral control. Although type I interferons are essential for early antiviral defense, prolonged signaling—particularly in the context of advanced aging—has been linked to endothelial activation, platelet priming, and immune-mediated tissue injury. Age-associated dysregulation of inflammatory and regulatory cytokine networks, including imbalances between IL-1 and IL-6 signaling and insufficient counter-regulation by IL-27 and IL-1 receptor antagonist, may further amplify this effect (43). Together, these findings support a model in which attenuated adaptive regulation coupled with persistent innate inflammatory signaling predisposes advanced-aged tissues to immunopathology and coagulopathy without conferring additional benefit in viral clearance.

Critically, coagulopathy was unique to advanced aged mice, indicating that it could be another focus of therapeutic intervention. Notably, coagulative dysfunction in severe SARS-CoV-2 cases has been clinically observed (44), and with the data provided in this manuscript, treatments such as heparin could be particularly relevant (45). It should be noted that multiple coagulation genes were up- and down- regulated, however, and intervention may also be similarly complex in addressing pathology. Importantly, these inflammatory and coagulatory pathways have significant overlap (46), and a combination of therapies could be investigated, with preclinical modeling being particularly important in allowing for separation and investigation of variables and for determining synergy in interventions.

These data, especially when considering correlations made between mice and humans, should offer resolution of previous publications from others showing contrasting data on inflammatory outcomes in aged mice, though others have provided their own data that could offer potential explanation to these contradictions, like the inoculum volume used in infecting mice (47) or the strains used (both mouse and virus), which our data should also be contextualized with. Most published studies used 16+ months of age as inclusion criteria for aged mice (though some have used younger), while our data show clear differentials in baseline and immune responses to viral infection between 15–18 month and 20+ month old mice, which could have been a contributing factor to the previously noted contradictory data. Enhanced pathology (both immune mediated and coagulation driven) in aged mice has been noted in other models as well, such as with LPS challenge (22) and even with SARS-CoV vaccination (48–50), though mouse strain (and T cell response skewing, ie. Th1 vs Th2) are important considerations when interpreting these studies (51).

There are several limitations of our studies that should be considered when interpreting the results. All mice used were female, which could bias the data presented towards sex related immune effects, especially considering the sex linked pathologies involved with SARS-CoV-2 infection (52). However, the human RNAseq data presented did incorporate males and females, and commonalities between mice and humans were still able to be made, partially alleviating this concern. Mouse weight was also not focused on, and mice do generally gain weight/adipose tissue like humans do while aging. This would be particularly relevant given the correlation between obesity and COVID outcomes (53), with many of the pathways of immune dysregulation between aging and obesity overlapping.

Another limitation of our approach was comparing influenza and MCMV infection in mice, to SARS-CoV-2 infection in humans. This was due to the difficulty of modeling SARS-CoV-2 infection in mice, with pathology being much less severe in mice due to differences in homology between mouse and human ACE2, the main binding target for SARS-CoV-2 (54). While advanced aged K18 hACE2 transgenic mice could be utilized in follow up SARS-CoV-2 studies, the overexpression of hACE2, and the co-expression of mACE2, makes pathology markedly worse in these mice than in humans (55–57). Because organ expression of hACE2 in these mice differs from humans, pathology based on artifacts in the model, such as severe neuropathology in infected mice, which is not fully representative of that seen in humans (58, 59), could be extrapolated and misapplied to the human scenario. While mouse adapted strains of COVID could circumvent this limitation, data availability is still limited, and differences in tropism and immune response have already been observed to exist when compared to human infection (60). Therefore, we chose an approach utilizing two distinct viruses encapsulating the components of SARS-CoV-2 infection, both respiratory and systemic. As SARS-CoV-2 preclinical modeling advances, our work should be revisited with these models as this would perhaps allow more direct conclusions regarding correlations between mouse and human acute viral immune mediated pathology, especially as investigation of the above-mentioned interventions is conducted.

While these data do not establish direct causal links between specific immune pathways and coagulopathy, they provide a mechanistic framework in which advanced age predisposes to immune-mediated vascular dysfunction during viral infection. This framework integrates inflammatory signaling, impaired anticoagulant regulation, and age-associated loss of immune resolution, and offers testable hypotheses for future studies aimed at dissecting immune–vascular interactions that drive severe outcomes in older hosts.

In conclusion, the data presented indicates that age induced immune dysfunction intensifies with advanced aging, resulting in decreased adaptive T cell responses concordant with increased innate and inflammatory pathways. The evidence provided that organ pathology is amplified, despite young, aged, and advanced aged mice having similar levels of viral control, would implicate immune pathology and coagulopathy as major drivers in the mortality that was observed in the studies. While both adaptive and innate immune pathways coordinate in host anti-viral responses, in younger recipients this leans towards adaptive responses which, being more specific and tightly regulated, aid in the control of immunopathology. Advanced age tips the scale towards innate (such as NK cells) and existing memory pathways, correlating with increased inflammation, pathology, and mortality.

## Data Availability

The datasets presented in this study can be found in online repositories. The names of the repository/repositories and accession number(s) can be found below: https://www.ncbi.nlm.nih.gov/geo/, PRJNA1293458.
