# Supplementary figures and images for: Age-driven shifts in T and NK cell responses amplify inflammation and coagulopathy during viral infection in mice and humans
Source: Front Immunol. 2026 Feb 9;17:1712726. doi: 10.3389/fimmu.2026.1712726 (PMC12926148; doi:10.3389/fimmu.2026.1712726)

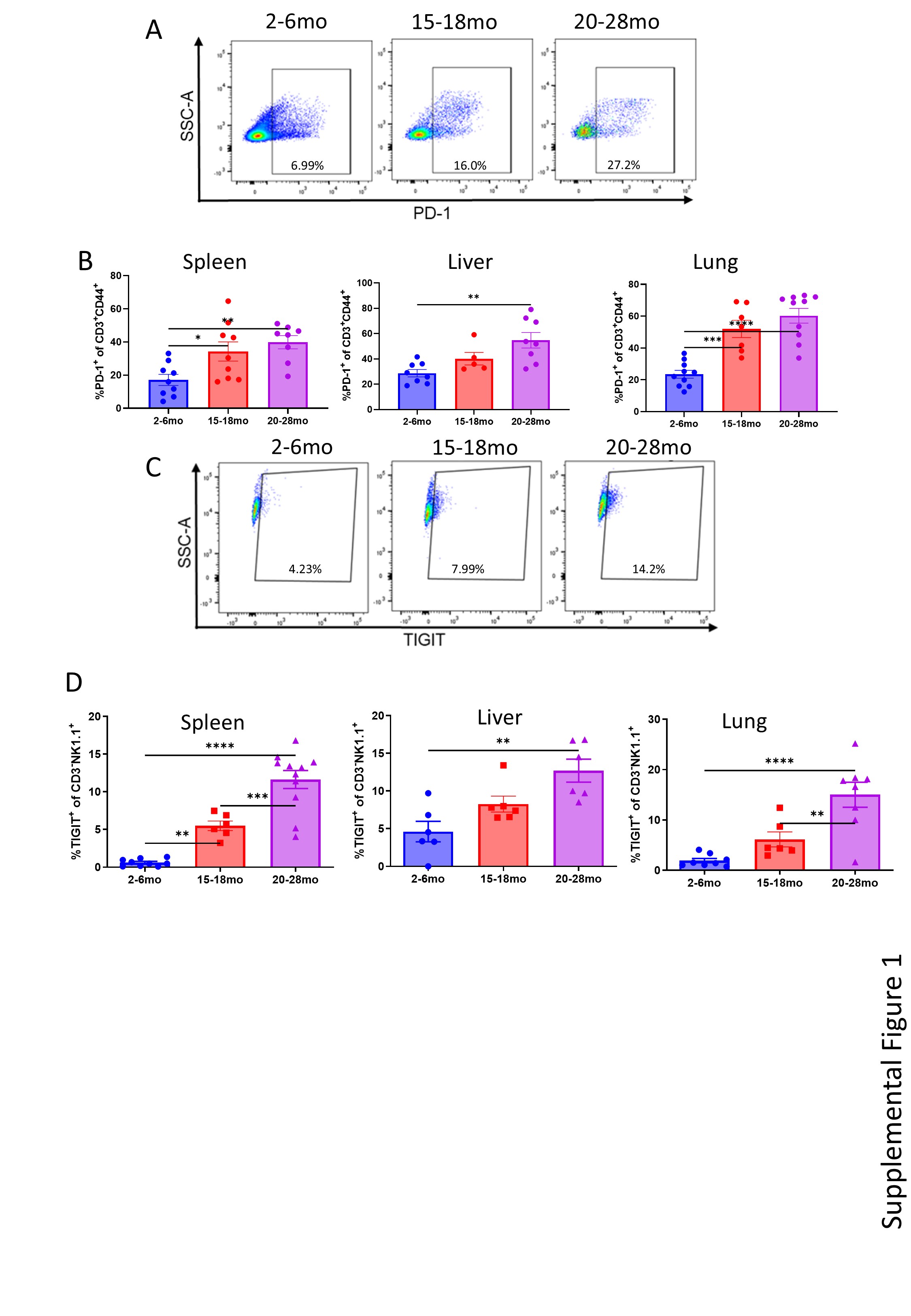

Supplement: Supplementary file 1 [file Image1.jpeg]

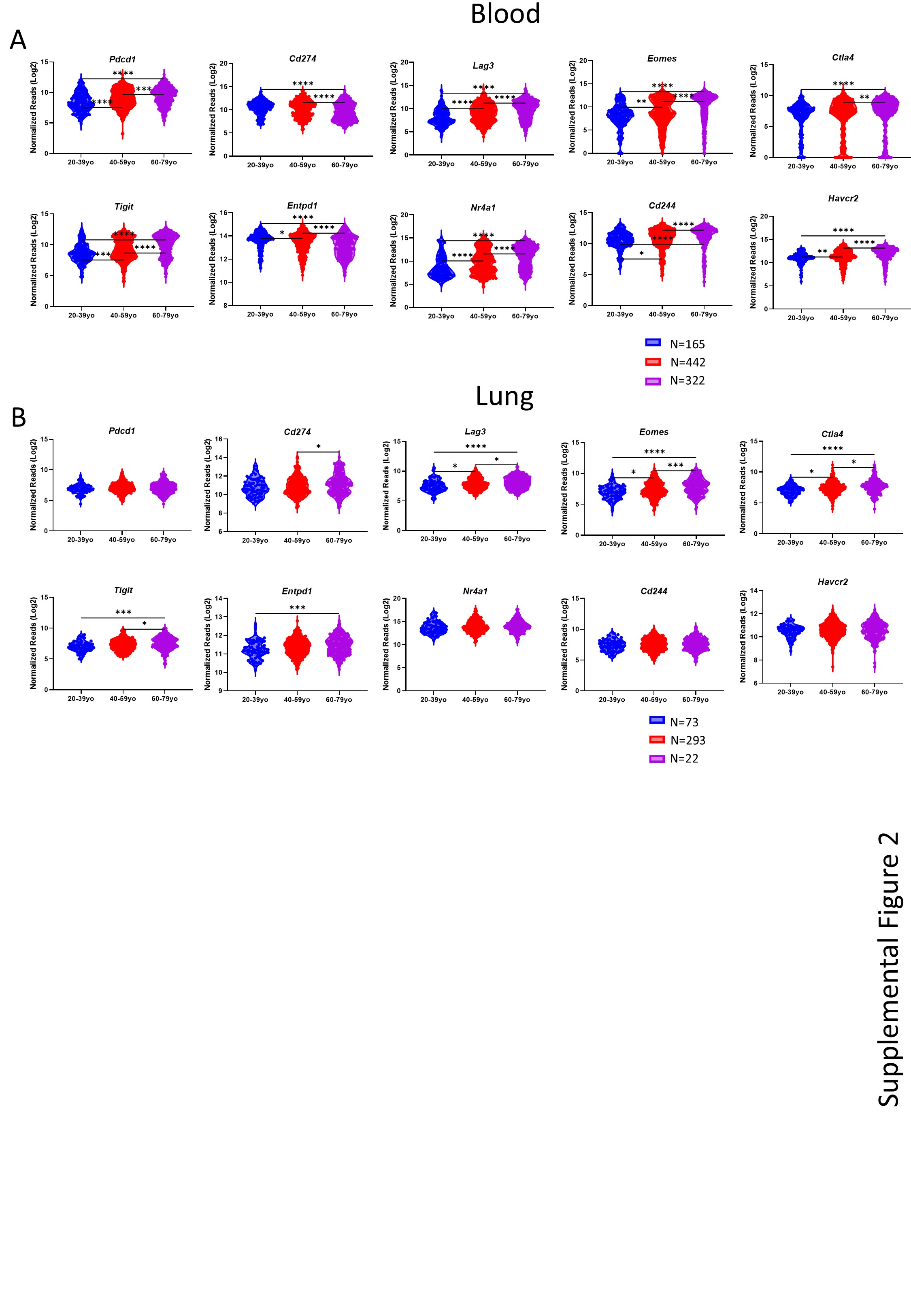

Supplement: Supplementary file 2 [file Image2.jpeg]

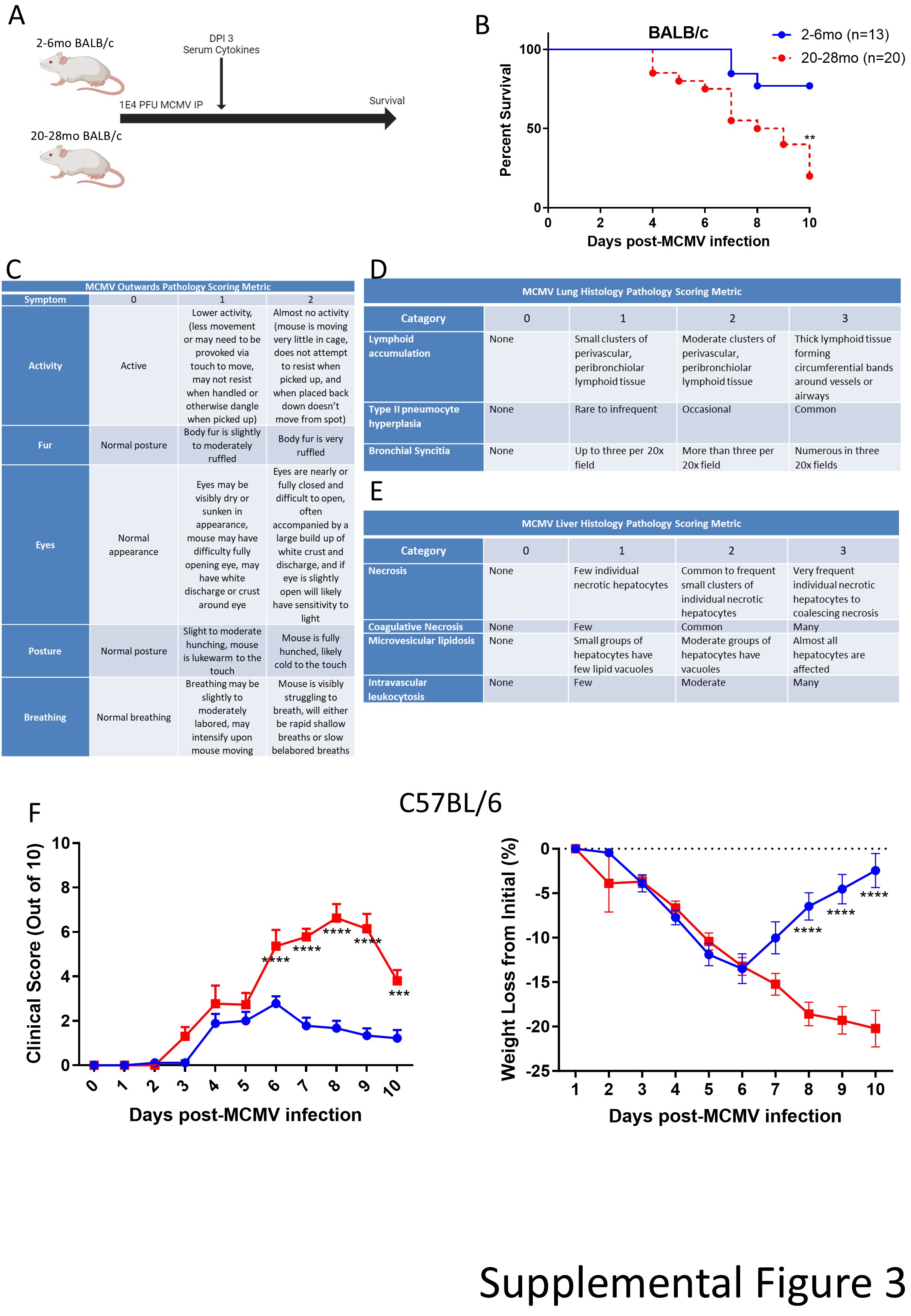

Supplement: Supplementary file 3 [file Image3.jpeg]

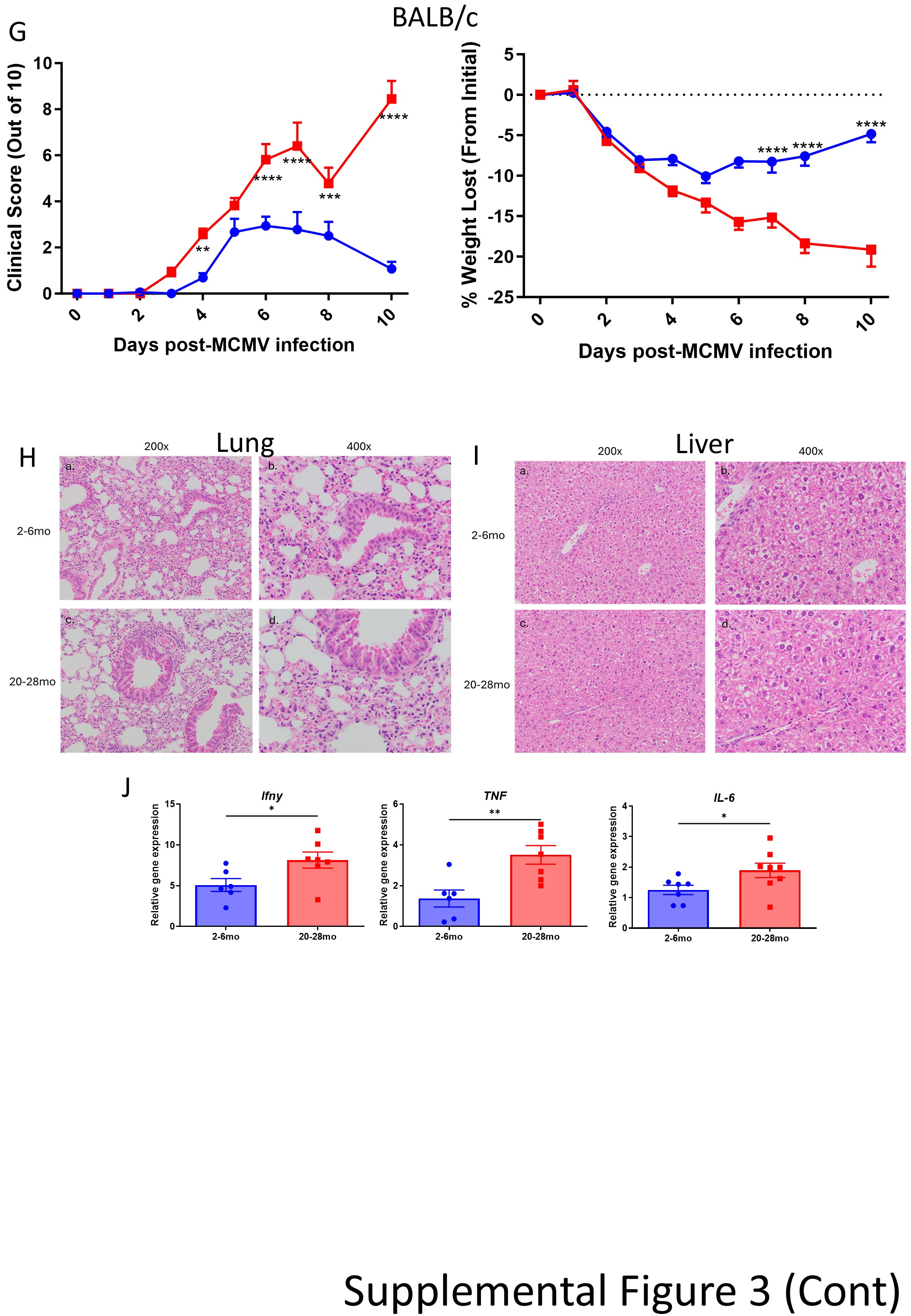

Supplement: Supplementary file 4 [file Image4.jpeg]

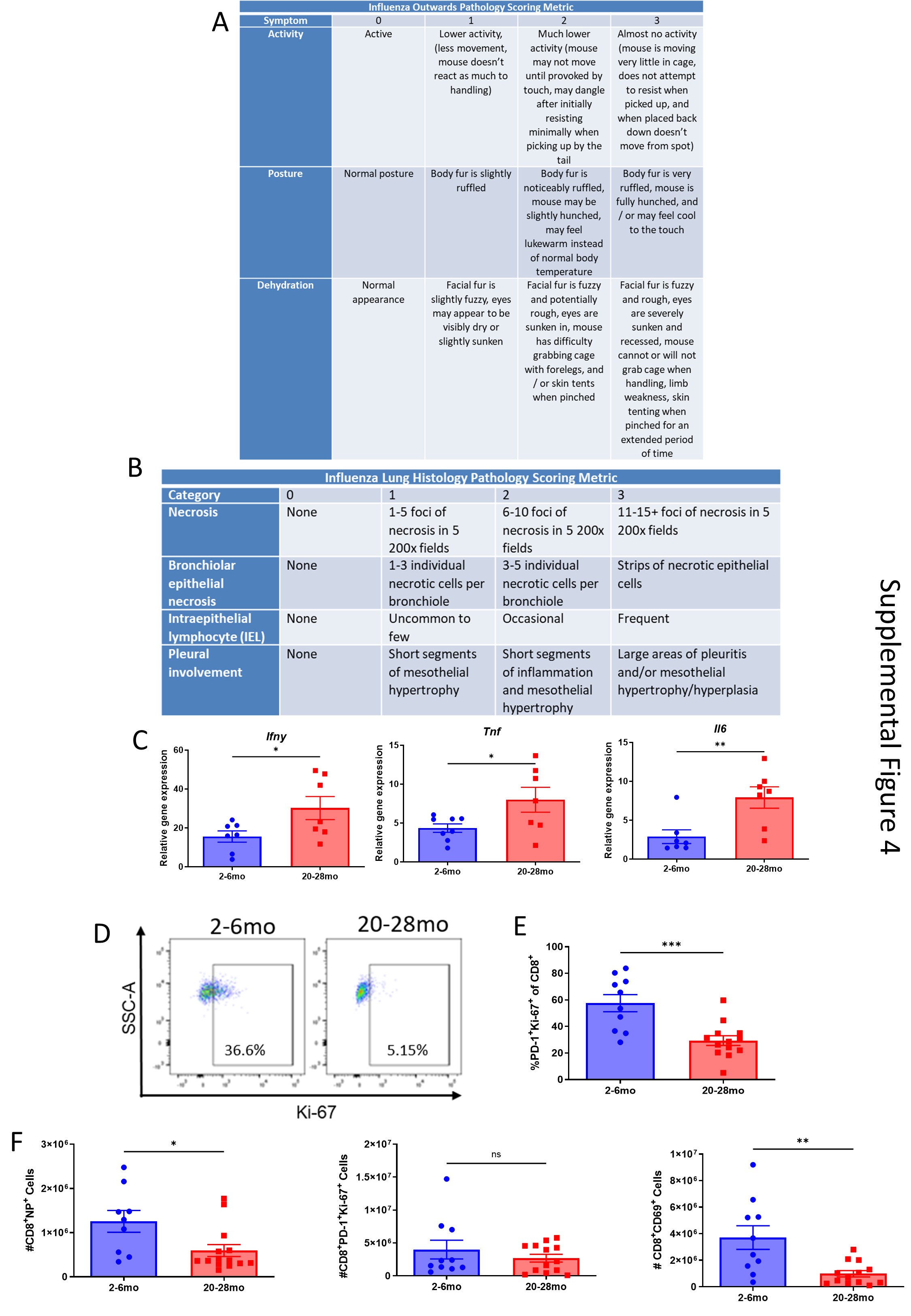

Supplement: Supplementary file 5 [file Image5.jpeg]

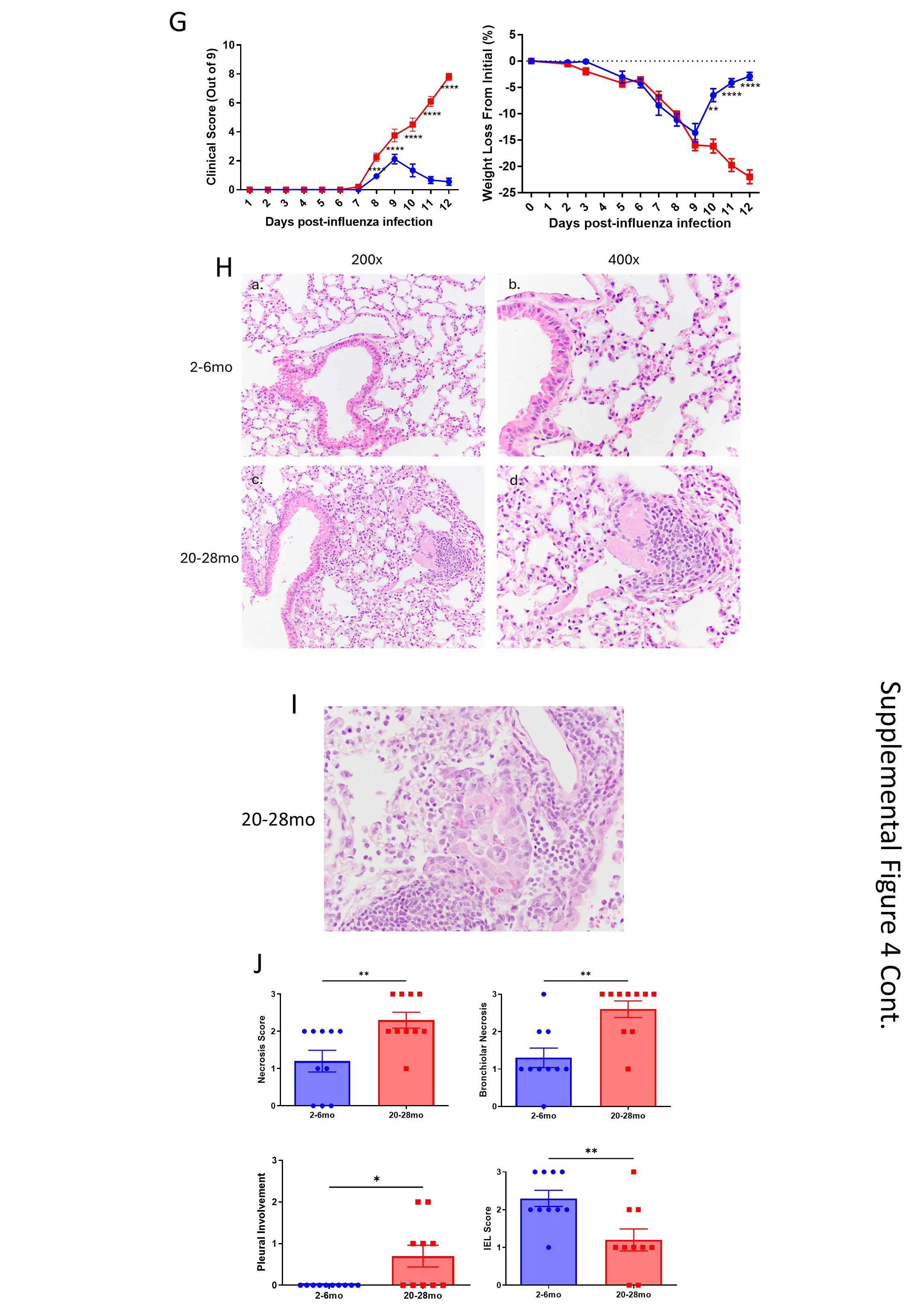

Supplement: Supplementary file 6 [file Image6.jpeg]

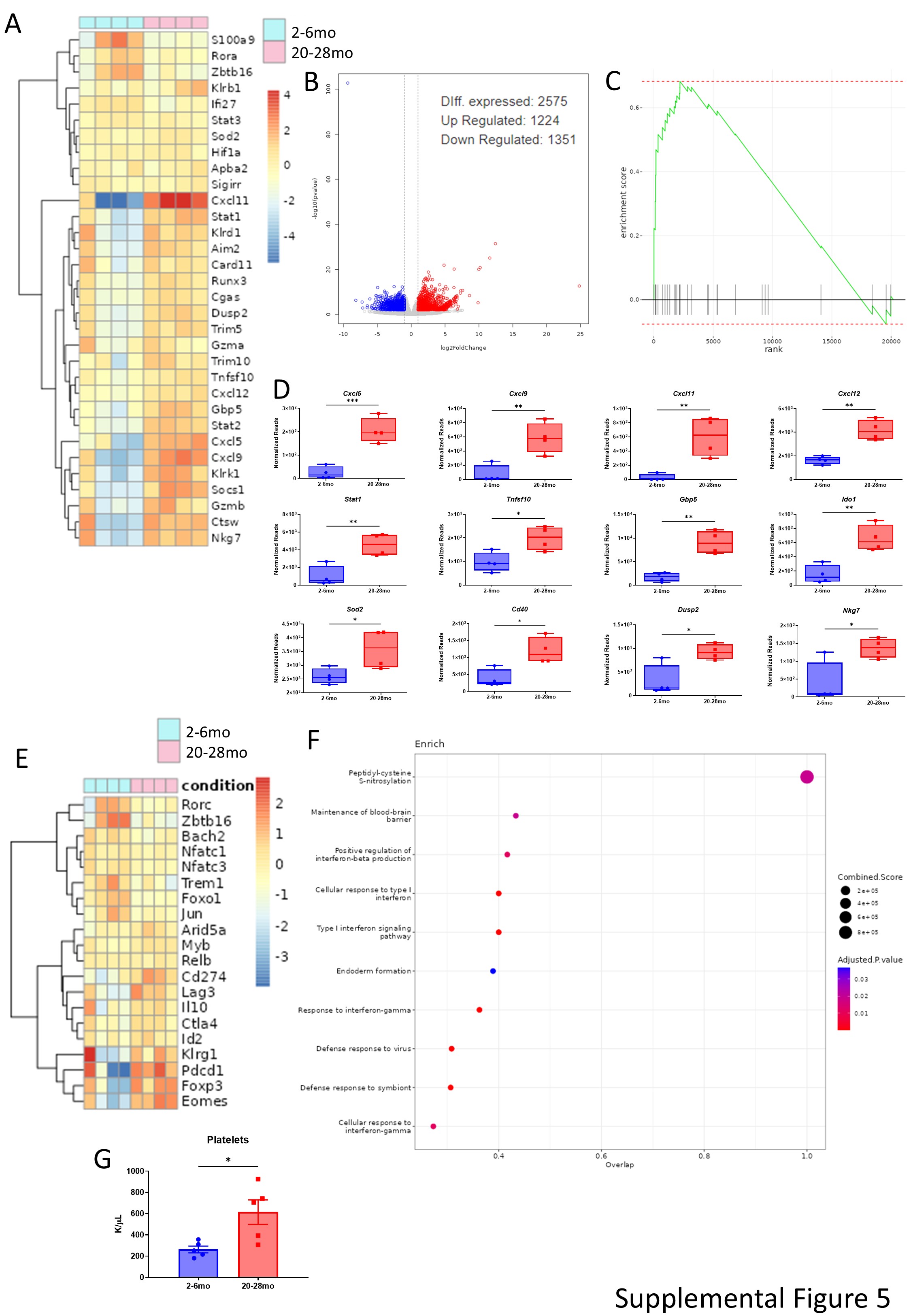

Supplement: Supplementary file 7 [file Image7.jpeg]

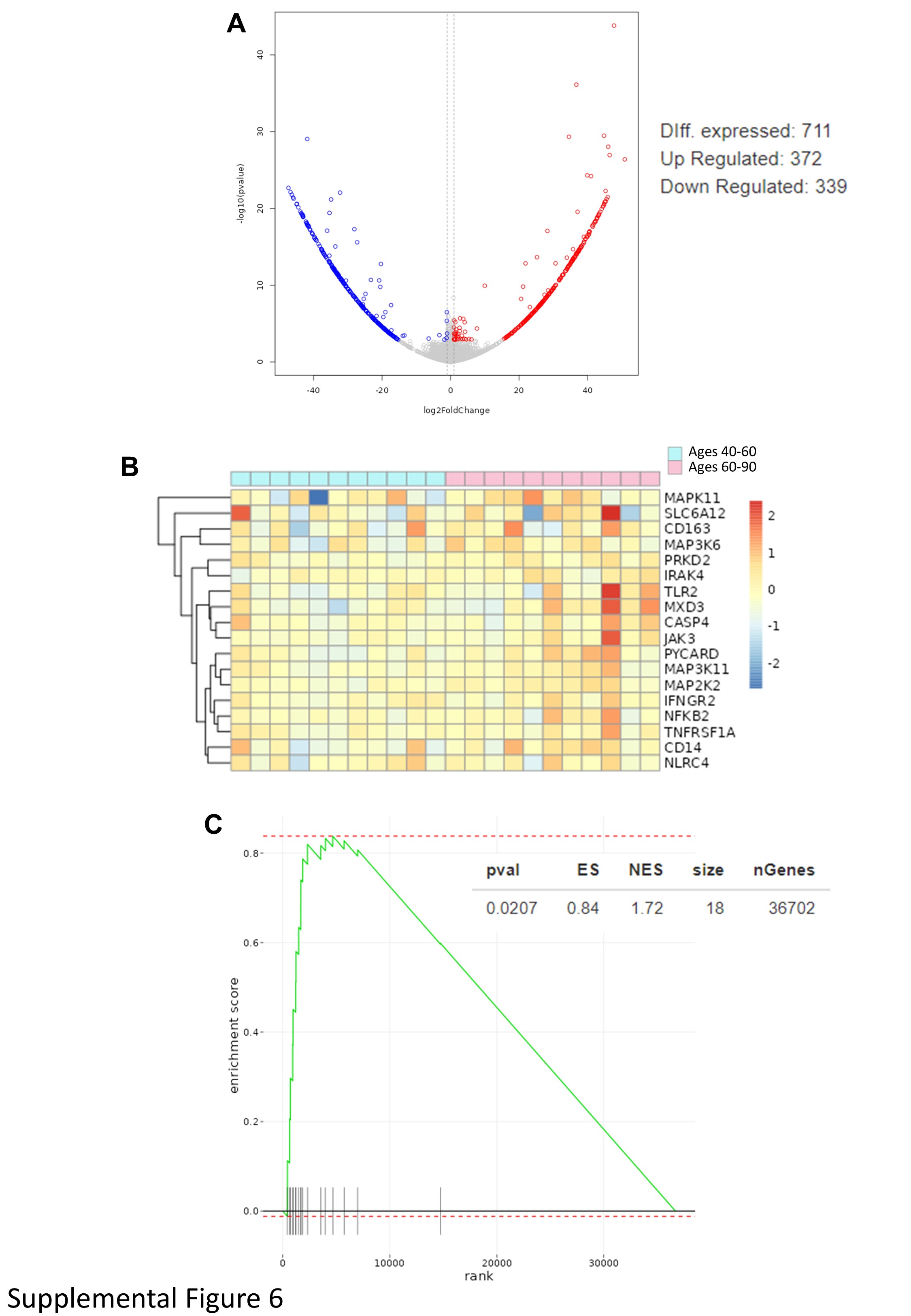

Supplement: Supplementary file 8 [file Image8.jpeg]
